# Defect in *BrMS1*, a PHD-finger transcription factor, induces male sterility in ethyl methane sulfonate-mutagenized Chinese cabbage (*Brassica rapa* L. ssp. *pekinensis*)

**DOI:** 10.3389/fpls.2022.992391

**Published:** 2022-08-18

**Authors:** Shiyao Dong, Jiaqi Zou, Bing Fang, Ying Zhao, Fengyan Shi, Gengxing Song, Shengnan Huang, Hui Feng

**Affiliations:** Department of Horticulture, Shenyang Agricultural University, Shenyang, China

**Keywords:** Chinese cabbage, male sterility, allelic mutants, PHD-finger transcription factor, RNA-seq

## Abstract

Male sterility is an ideal character for the female parent in commercial hybrid seed production in Chinese cabbages. We identified three allele male sterile mutants *msm2-1*/2/3 in progenies of ethyl methane sulfonate mutagenized Chinese cabbage. It was proved that their male sterilities were controlled by a same recessive nuclear gene. Cytological observation showed that the delayed tapetal programmed cell death (PCD) as well as the abnormal pollen exine and intine led to pollen abortion in these mutants. MutMap combined with KASP analyses showed that *BraA10g019050.3C*, a homologous gene of *AtMS1* encoding a PHD-finger transcription factor and regulated pollen development, was the causal gene. A single-nucleotide mutation from G to A occurred at the 2443th base of *BrMS1* in *msm2-1* which results in premature termination of the PHD-finger protein translation; a single-nucleotide mutation from G to A existed at 1372th base in *msm2-2* that makes for frameshift mutation; a single-nucleotide mutation from G to A distributed at 1887th base in *msm2-3* which issues in the amino acid changed from Asp to Asn. The three allelic mutations in *BrMS1* all led to the male sterile phenotype, which revealed its function in stamen development. Quantitative reverse transcription polymerase chain reaction analysis indicated that *BrMS1* specially expressed in the anther at the early stage of pollen development and its expression level was higher in *msm2-1/2/3* than that in the wild-type “FT.” BrMS1 was located at the nucleus and a length of 12 amino acid residues at the C-terminus had transcriptional activation activity. RNA-seq indicated that the mutation in *BrMS1* affected the transcript level of genes related to the tapetum PCD and pollen wall formation, which brought out the pollen abortion. These male sterile mutants we developed provided a novel gene resource for hybrid breeding in Chinese cabbage.

## Introduction

Male sterility refers to the phenomenon in which stamens are degenerated or malformed, and viable male gametes are not produced, while female gametes are fertile in flowering plants. Male sterility has been widely used for hybrid seed production (Fan and Zhang, 2018). Up to now, many genic male sterility (GMS) genes have been identified in plants (Shi et al., 2015; Wan et al., 2019, 2020), and a great deal of effort has been made to develop male-sterility systems by using these GMS genes and their corresponding mutants to maintain and propagate GMS lines as female parents for hybrid seed production (Kim and Zhang, 2018). The male sterility system is also of immense significance in probing the molecular mechanism of pollen development. Spontaneous mutant male sterile mutants have been widely used in functional genomics (Liu et al., 2016; Liang et al., 2017; Zhou et al., 2017; Lin et al., 2020; Zhang et al., 2020). Due to its scarcity, artificial mutagenesis including physical mutagenesis, chemical mutagenesis, biological mutagenesis, and other methods to create male sterile mutants, has become an important measure for the study of gene regulatory networks for male gamete development (Ma et al., 2019; Tan et al., 2019; Huang et al., 2020).

Male gamete formation in anthers requires the coordinated participation of both sporophytic and gametophytic tissues (Hafidh et al., 2016). The tapetum, the innermost cell layer of the anther wall, plays a crucial role in regulating pollen development. The tapetum cells could provide the nutrients required for the meiosis of the microspore mother cell and the development of the microspore (Wu and Cheung, 2000; Parish and Li, 2010). During the tetrad period, the tapetum secrete sporopollenin precursor substances and they deposit continuously on the primexine to form a special T-shaped structure composed of the tectum, bacula, and foot layer (Shi et al., 2015). After the microspore mother cell completes meiosis, the tapetum synthesizes callose enzymes to degrade callose, which promotes the release of independent uninucleate microspores (Zhang et al., 2007; Parish and Li, 2010). Then, the tapetum undergoes programmed cell death (PCD), the *pollen coat proteins* (*PCPs*) and the tapetal cell debris produced by tapetum degradation, fill the pollen exine to form the pollen coat (Zhang et al., 2016). Therefore, abnormalities at any stage of the tapetum development could cause pollen aberrations.

The development of the tapetum is a precise and orderly process, which is jointly regulated by a variety of genes. To date, there are three major types of transcription factors (TFs) that regulate the development and function of the tapetum: *bHLH* TFs such as *DYT1* and *AMS*, *MYB* TFs such as *TDF1* and *MS188*, and *PHD-fingers* TFs such as *MS1* and *TIP3* (Zhu et al., 2008, 2011; Zhang and Yang, 2014; Wan et al., 2019; Yang et al., 2019a). These TFs cooperatively form the tapetal genetic pathway *(DYT1-TDF1-AMS-MS188-MS1)* and strictly control tapetum development (Zhu et al., 2011). Several orthologs of *MS1* have been identified in different species. In *Arabidopsis*, *AtMS1* directly regulated the expression of multiple *PCPs* such as *GRP14*, *GRP19* and affected the expression of *KCS7*, *KCS15*, and *KCS21* for *pollen coat lipids* (*PCLs*) synthesis (Lu et al., 2020; Zhang et al., 2021). In *rice*, *OsMS1/OsPTC1* can interact with *OsMADS15* and *TIP2*. It has been reported that *TIP2* coordinated with TDR to modulate the expression of *EAT1* and *Cys* protease gene (*CP1*) further regulated tapetal PCD and pollen exine formation in rice (Li et al., 2011; Yang et al., 2019b). In *maize, ZmMs7* can interact with maize *nuclear factor Y (NF-Y)* subunits to form *ZmMs7-NF-YA6-YB2-YC9/12/15* protein complexes that activate target gene *ZmMT2C* to regulate the tapetal development (An et al., 2020). However, the molecular mechanism by which the homologous gene of *MS1* in Chinese cabbage affects pollen development remains unclear.

Chinese cabbage (*Brassica rapa* L. ssp*. pekinensis*) is one of the most important vegetable crops in eastern Asia, which is a typical cross-pollinated *Brassica* crops with obvious heterosis (Sharma et al., 2021). Breeding of male sterile lines is crucial for the commercialization of Chinese cabbage hybrid seed production (Singh et al., 2019). Mapping and cloning the male sterile genes are preconditions to the study of its molecular mechanism and applications. The genic male sterile lines could be created by artificial mutagenesis in Chinese cabbage. Tan et al. (2019) developed a male sterile mutant (*ftms*) from Chinese cabbage DH line “FT” by irradiating microspores with ^60^Co γ-rays while *Bra010198* was a candidate gene for male sterile mutant (*ftms*), which encoded a putative *β-(1,3)*-galactosyltransferase that controls pollen exine development. Huang et al. (2020) obtained a stably inherited male sterile mutant (*msm*) by the identical methods and compared the differential expression genes of “FT” and *msm* flower buds using RNA-Seq technology.

Here, we identified three allele male sterile mutants named *msm2-1*/2/3 in an ethyl methane sulfonate (EMS)-mutagenized Chinese cabbage progenies. Cytological observation revealed delayed tapetum PCD, defective pollen exine and intine formation in *msm2-1* mutant. We discovered by MutMap and KASP that the non-synonymous base-pair mutations in *BrMS1* (*BraA10g019050.3C*) induced the pollen abortion in the allele mutants *msm2-1-2/3*. *BrMS1* was a homologous gene of *AtMS1*, which encoded a PHD-finger TF and regulated the pollen development. The protein encoded by the *BrMS1* was located at the nucleus in Chinese cabbage. Quantitative reverse transcription polymerase chain reaction (qRT-PCR) performed that *BrMS1* expressed specifically in the anther at the early stage of pollen development and its expression level was higher in *msm2-1/2/3* versus in the wild-type “FT.” The analysis of RNA-seq exposed that the expression level of genes associated with the tapetum PCD and pollen wall synthesis has changed significantly in the mutant *msm2-1*. Our research is the first to clone an *AtMS1* orthologs gene relevant to fertility regulation in Chinese cabbage, thereby providing basal information for applying this critical agronomic trait in hybrid breeding and studying the molecular mechanism of pollen development.

## Materials and methods

### Plant materials

The Chinese cabbage doubled haploid (DH) line “FT,” obtained from a microspore culture of the commercial variety “Fukuda 50,” was used as the wild type. We treated “FT” seeds with 0.8% EMS, the live plants (M_0_) were self-pollinated, M_1_ seeds were harvested. After M_1_ self-pollination, we obtained stably inherited mutations in the M_2_ generation (Gao et al., 2022). Among them, nine male sterile mutants with stable inheritance and fertility not affected by environment were identified after multiple generations phenotypic identification (Supplementary Figure S1). The fertile and sterile plants in AB lines of the nine male sterile materials were performed the allelic test by mutual cross. The materials whose segregation ratio of the hybrid progeny conformed to 1:1 were allelic mutants of each other (Supplementary Table S1). We screened out two groups of allelic mutant materials in total and select one group (*msm2-1*, *msm2-2*, and *msm2-3*) as the experimental materials for this study. All plant materials were cultured in a greenhouse at 20–28°C in Shenyang Agricultural University (Shenyang, P. R. China).

### Genetic analysis

For the genetic analysis, the wild-type “FT” (P_1_) was crossed with *msm2-1/2/3* mutants (P_2_) to generate the F_1_ and F_2_ populations. Fertile plants and sterile plants from the F_2_ population were selected for crossing to generate AB line. Phenotypic characterization was obtained for each generation (P_1_, P_2_, F_1,_ F_2_ and AB line), and the segregation ratio of the population was analyzed by the Chi-square test.

### Morphological observation of floral organs

To observe the difference in floral organs between the wild-type “FT” and *msm2-1/2/3*, the five floral organs (sepals, petals, filament, stamens, pistils) were collected at the full-bloom stage and dissected under a stereomicroscope (Nikon SMZ800, Japan). For investigating the pollen vitality, the fresh anthers at the full-bloom stage were extruded and immersed in 0.1% 2,3,5-triphenyltetrazolium chloride dye solution on a carrier plate covered with a coverslip. Pollen viability was analyzed *via* applying an optical microscope (Nikon ECLIPSE 80i, Japan).

### Anther cytological observation

For understanding more about the period of pollen abortion, the flower buds of the wild-type “FT” and *msm2-1/2/3* were fixed using FAA solution (50% ethanol, 5% glacial acetic, 10% formalin), then dyed using safranin and fast green, and observed under an optical microscope (Nikon ECLIPSE 80i; Nikon, Japan). The buds were divided into six grades according to the length of the buds (BUD1 < 1.5 mm, BUD2: 1.5–2.0 mm, BUD3: 2.0–2.5 mm, BUD4: 2.5–3.0 mm, BUD5: 3.0–3.5 mm, BUD6 > 3.5 mm.). The detailed paraffin sections operation steps were performed according to the method described by Zhou et al. (2017).

For scanning electron microscopy (SEM), to observe the morphology of anther and pollen exine, the fresh anthers of the wild-type “FT” and the *msm2-1* mutant were collected at the full-bloom stage and fixed in 2.5% glutaraldehyde solution at 4°C for 48 h, processed according to the methods of Lin et al. (2014) and then examined under SEM (Hitachi TM3030, Japan).

For transmission electron microscopy (TEM), to further explore the morphology changes of pollen exine, the anthers are roughly divided into three periods: the early stage (BUDS < 2.0 mm), middle stage (2.0 mm < BUDS < 3.0 mm), and the late stage (BUDS > 3.0 mm) according to the pollen development process. Anthers at different pollen developmental stages from “FT” and *msm2-1* plants were examined by TEM (Hitachi Ltd., Tokyo, Japan) after a series of treatments as previously described (Wang et al., 2020).

### Screening of the candidate genes by MutMap

The candidate gene was identified by the modified MutMap method. Fifty plants with mutant phenotype were selected from the F_2_ plants, and a DNA pool was constructed by pooling equal amounts of plant leaf tissue from each plant. DNA was acquired from the wild-type “FT” and *msm2-1* mutant, the DNA pool was extracted using a DNA-secure Plant Kit (Tiangen, Beijing, China) and was re-sequenced using a NovaSeq 6,000 System sequencer (Illumina, San Diego, United States). The analysis method was the same as described by Gao et al. (2020) and Wang et al. (2020).

### SNP genotyping through KASP

KASP was applied for the genotypic assay to detect the co-segregation of each single nucleotide polymorphism (SNP) identified by MutMap and confirmed the candidate gene. Allele-specific primers bearing the FAM and HEX fluorescence probes and the common primer were designed by LGC (Laboratory of the Government Chemist, Shanghai, China) and used to determine the male sterile genotypes of 183 F_2_ plants (Supplementary Table S2). The primer mixed and PCRs were programmed as recommended by LGC. The KASP thermal cycling conditions were those described in Xi et al. (2018). Fluorescence was detected with a QuantStudio 6 instrument (Applied Biosystems, Foster City, CA, United States).

### Cloning and sequencing of the candidate gene

The full-length and CDS sequences of candidate genes were obtained by cloning and sequencing. Primers were designed according to the gene sequence information and are shown in Supplementary Tables S3, S4. We purified the PCR products with a Gel Extraction Kit (CWBIO, Beijing, China) and then introduced the purified products into a pGEM-T-Easy Vector (Promega, United States). The products were sequenced at GENEWIZ (Suzhou, China). We analyzed the sequences using DNAMAN V6 (Lynnon BioSoft, Canada).

### Phylogenetic analysis and bioinformatic analysis of BrMS1 protein

The sequence and the information of BrMS1 were downloaded from the Brassica database. BrMS1 protein homologs in various plants were obtained from the GenBank database^1^ and aligned with ClustalW (Larkin et al., 2007). A phylogenetic tree was constructed with MEGA6.0 by the neighbor-joining (NJ) method based on a bootstrap test of 1,000 replicates (Tamura et al., 2013). The conserved domain of BrMS1 was analyzed online at NCBI.^2^

### Expression patterns analysis by qRT-PCR

Total RNA was isolated from different tissues of the wild-type “FT” and *msm2-1/2/3* at developmental stages with the TIANGEN RNA prep Pure Plant Kit. First-strand cDNA was synthesized using Fast Quant RT SuperMix (Tian gen). The cDNA was subsequently used as a template for qRT-PCR together with SYBR Green PCR Master Mix (TaKaRa, Dalian, China). ACTIN was used as the internal reference gene. Specific primers for each target gene are listed in Supplementary Table S5. Each sample had three biological replicates, each with three technical replicates.

### Subcellular localization of BrMS1 proteins

To generate the *BrMS1–GFP* fusion protein, the full-length CDS of *BrMS1* was amplified. The coding regions of the *BrMS1* sequences were amplified without stop codons using the primers *BrMS1-GFP-F* and *BrMS1-GFP-R*. They were cloned into the C-terminal GFP fusion vector *pBWA(V)HS* driven by the *35S* promoter. Vector-bearing, *35S*-driven GFP was the negative control. Subcellular localization of the *BrMS1* proteins was performed according to Yoo et al. (2007). The constructs were then transiently transformed into tobacco (*Nicotiana benthamiana*) leaves through Agrobacterium tumefaciens infiltration. After 48 h, the epidermis of the tobacco leaves was injected with *DAPI* and examined with a confocal laser-scanning microscope (Leica Microsystems, Wetzlar, Germany). The primer sequences used in this experiment are shown in Supplementary Table S6.

### Transactivation activity assay

The transcriptional activity assay was performed using the Matchmaker GAL4 Yeast Two-Hybrid System 3 (Clontech). To generate the BD–*BrMS1*, BD–ΔN, BD–ΔC and BD–PHD constructs, the full-length CDS and various deletions of *BrMS1* were PCR amplified and inserted into the pGBKT7 plasmid linearized. All recombinant plasmids and a pGBKT7 empty vector were separately transformed into the Y2H Gold Yeast strain. Then they were initially grown on the medium lacking Trp for 3 days at 30°C. Four of the resulting independent colonies were transferred onto the medium without Trp, His, and Ade for 3 days at 30°C to assay transcriptional activity. The primers used for vector construction are listed in Supplementary Table S7.

### RNA-seq

To identify transcripts involved in the regulation of pollen abortion in the Chinese cabbage, we performed an extensive transcriptomic analysis of entire anther. Total RNA was extracted from the wild-type “FT” and *msm2-1* with the RNA prep Pure Plant Plus Kit (Tiangen, Beijing, China). Samples were collected with three biological replicates. Six cDNA libraries (F1, F2, F3, S1, S2, and S3) were constructed and sequenced using the Illumina HiSeq™ 2,500 sequencing platform at Beijing Genomics of Institute (BGI), Shenzhen, China. The detailed method and procedure have been described previously by Huang et al. (2017). In this study, different expression genes (DEGs) were defined as those genes with a false discovery rate (FDR) ≤ 0.01 and an absolute log2 ratio value ≥ 10. To further study the biological functions and metabolic pathways of the DEGs, we performed Gene Ontology (GO)^3^ functional analysis and Kyoto Encyclopedia of Genes and Genomes (KEGG) pathway enrichment analysis of these genes (Ashburner et al., 2000; Kanehisa et al., 2007).

## Results

### Genetic analysis and morphological characteristics of the male sterile mutants *msm2-1/2/3*

To elucidate the genetics of the male sterile phenotype, the wild-type “FT” (P_1_) and the mutants *msm2-1/2/3* (P_2_) were employed as the male parent and the female parent to generate F_1_ and F_2_ populations. All F_1_ individuals displayed male fertile phenotype. As disclosed by the Chi-square test, the segregation rate of the F_2_ population corresponded to the expected Mendelian ratio of 3:1 (fertile:sterile; Table 1). Allelism test showed that the fertility separation ratio of *msm2-1*, *msm2-2* and *msm2-3* hybrid offspring was 1:1 (fertile:sterile), which implied that mutant *msm2-1*, *msm2-2* and *msm2-3* were controlled by an allelic gene (Table 2). Therefore, these results indicated that the male sterile trait of *msm2-1/2/3* mutants was controlled by a single nuclear recessive gene. From the perspective of breeding, it also demonstrated that allele gene mutation result in male sterility in the mutants *msm2-1/2/3.*

**Table 1:** Analysis for the genetic characteristics of the mutants *msm2-1/2/3.*

Generation	Total	Male ferility	Male sterility	Segregation radio	*χ* ^2^
P_1_(“FT”)	1	1	0		
P_2_(*msm2-1*)	1	0	1		
F_1_(P_2_×P_1_)	100	100	0		
F_2_	314	239	75	3.19:1	0.21
P_2_(*msm2-2*)	1	0	1		
F_1_(P_2_×P_1_)	100	100	0		
F_2_	122	96	26	3.69:1	0.89
P_2_(*msm2-3*)	1	0	1		
F_1_(P_2_×P_1_)	100	100	0		
F_2_	63	49	14	3.50:1	0.26

**Table 2:** Analysis for allelism test of the mutants *msm2-1/2/3.*

Generation	Total	Male ferility	Male sterility	Segregation radio	*χ* ^2^
*msm2-1* × *msm2-2*	50	23	27	0.85:1	0.32
*msm2-1* × *msm2-3*	50	26	24	1.08:1	0.08
*msm2-2* × *msm2-3*	49	25	24	1.04:1	0.02

**Figure 1:**
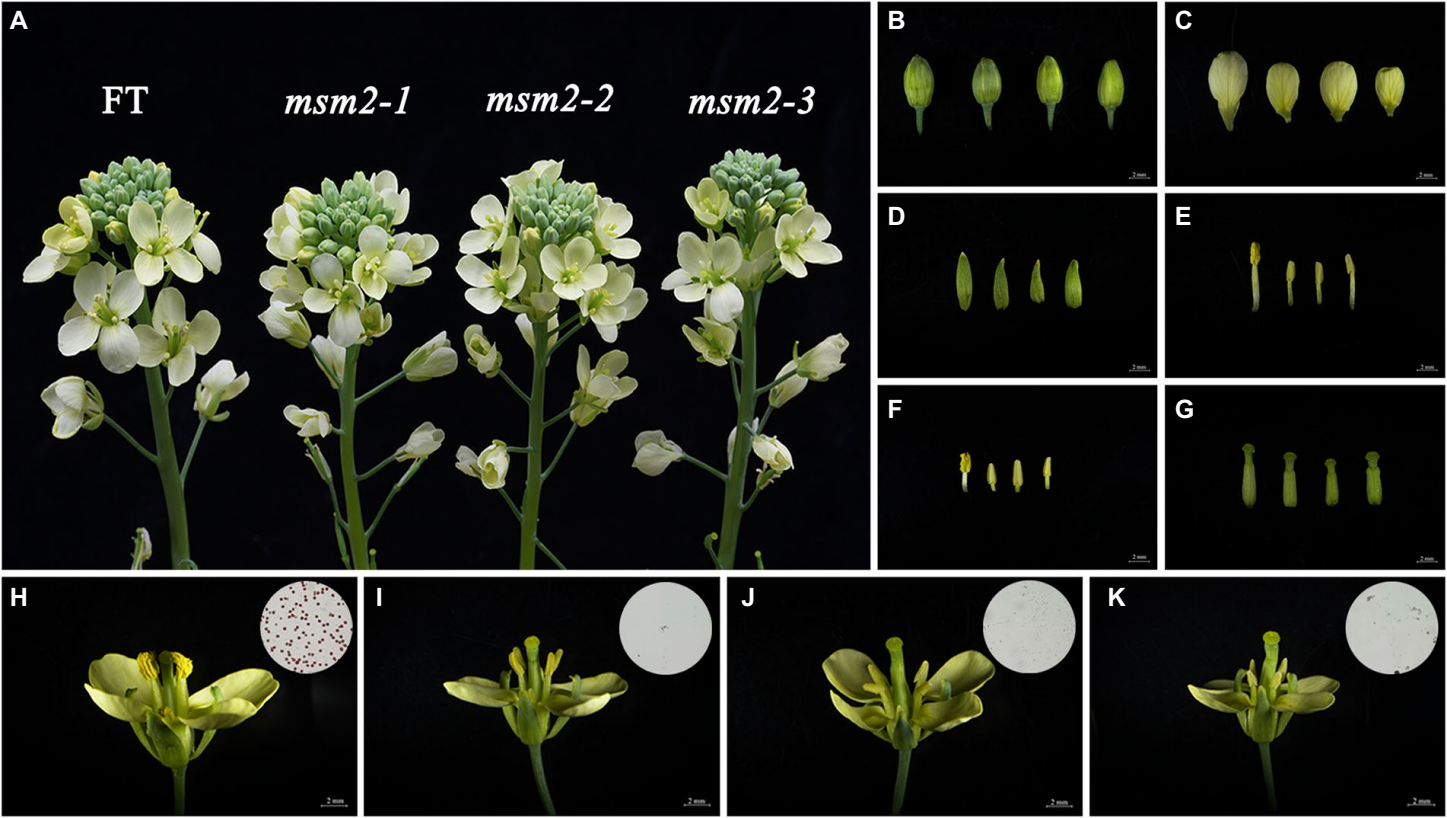
Phenotypic analysis of the wild-type “FT” and *msm2-1/2/3* mutants. Comparison of the floral organs of the wild-type “FT” and mutant *msm2-1/2/3* (from left to right). **(A)** Inflorescence **(B)** buds, **(C)** petals, **(D)** sepals, **(E)** stamens (long), **(F)** stamens (short), **(J)** pistils. Scale bar 2 mm analysis of pollen viability of the wild-type “FT” **(H)**, *msm2-1* **(I)**, *msm2-2* **(J)**, and *msm2-3* **(K)**. Scale bar 150 μm.

At the full-bloom stage, the morphology of floral organs was investigated. The stamens of *msm2-1/2/3* had developed normally yet no pollen attached to anthers (Figure 1A). Other flower organs in the *msm2-1/2/3* mutants were found to be slightly smaller than those in the wild-type “FT” (Figures 1B–G). Pollen viability observations also illustrated that the wild-type “FT” could generate mature and active pollen grains, whereas *msm2-1/2/3* could not produce pollen grains (Figures 1H–K). These three *msm2-1/2/3* mutants were allelic as evidenced by allelic test and manifested similar phenotypes under the same growth conditions; thus, some of the subsequent analyses in this study were only performed on *msm2-1*.

### Cytological observation of the anther development

**Figure 2:**
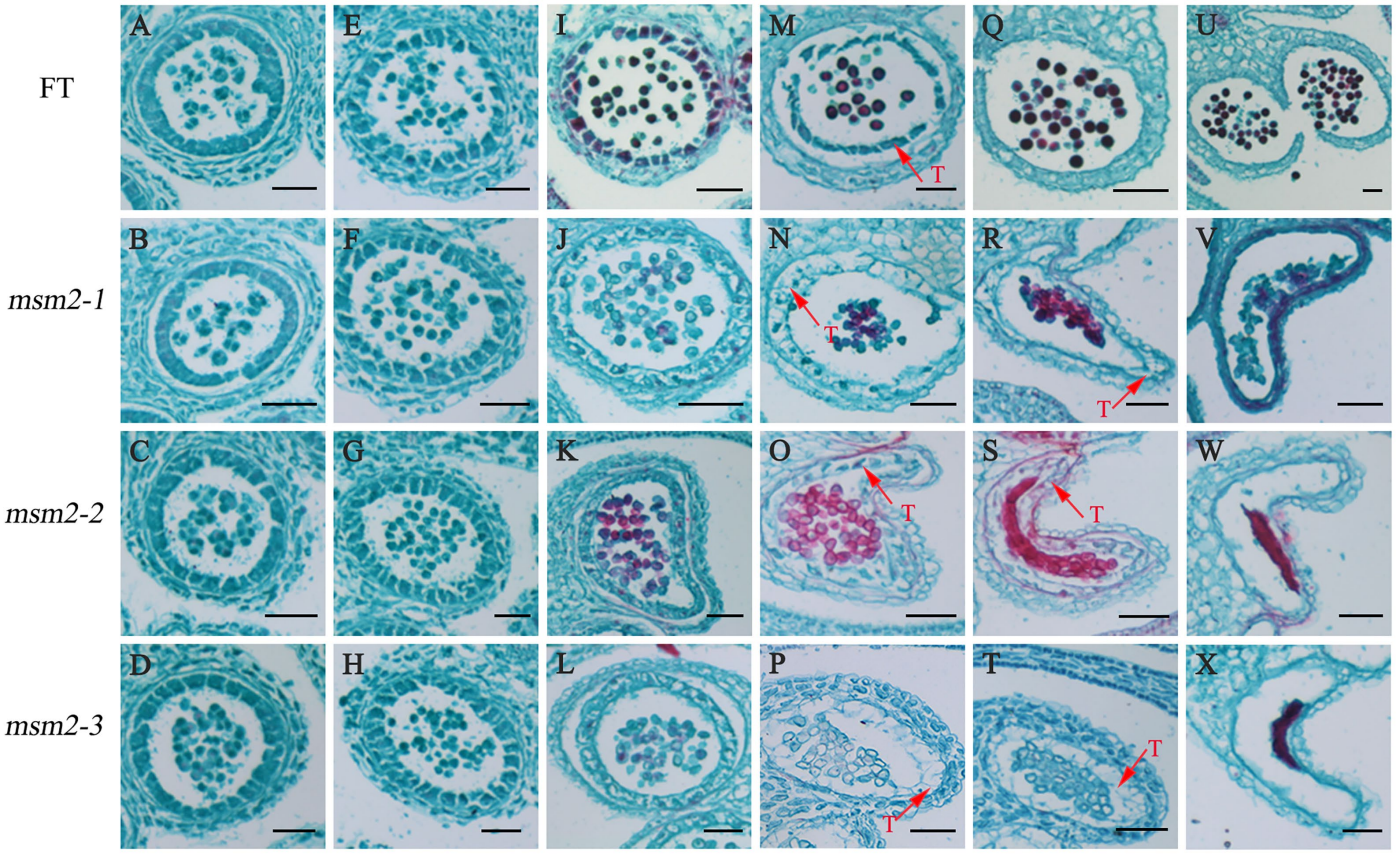
Anther paraffin section observation of the wild-type “FT” and *msm2-1/2/3* mutants. **(A–D)** The stage of tapetal formation (i.e., the tetrad period), BUD1 < 1.5 mm; **(E–H)** The stage of the tapetum cell structure integrity and clearly demarcated (i.e., the uninucleate microspore stage), BUD2: 1.5–2.0 mm; **(I–L)** The stage of tapetal cytoplasm dense, BUD3: 2.0–2.5 mm; **(M–P)** The stage of tapetum cells begin the degradation, BUD4: 2.5–3.0 mm; **(Q–T)** The stage of tapetum cells complete the degradation, BUD5: 3.0–3.5 mm; **(U–X)** The stage of anthers dehiscence and mature pollen release, BUD6 > 3.5 mm. T tapetum. Scale bar, 50 μm.

To explore the cytological characteristics of pollen abortion in *msm2-1/2/3*, we examined paraffin sections prepared at different anther development stages of the wild-type “FT” and the *msm2-1/2/3*. The paraffin section analysis showed there was no significant difference in *msm*2-*1/2/3* compared with the wild-type “FT” at the early stage of the anther development (Figures 2A–H). At the middle stage of anthers development, the wild-type tapetum was concentrated with less vacuolation and darker staining. However, the tapetum cells became more vacuolated and less stained in *msm*2-*1/2/3* (Figures 2I–L). Then, the wild-type tapetum cell appeared to undergo degeneration, and the cytoplasmic constituents remained densely stained. By contrast, we discovered that the tapetum PCD was retarded in the *msm2-1/2/3* mutants and the tapetum cells had vacuolated (Figures 2M–T). At the late stage of anthers development, the tapetal cells degenerated and the pollen grains matured with a round shape in the wild-type “FT.” Nevertheless, cell debris of tapetal cells and immature pollen grains remained in the *msm2-1/2/3* (Figures 2U–X). Paraffin sections of anther indicated that the degradation of tapetum cell delayed occurred in mutants *msm2-1/2/3*.

**Figure 3:**
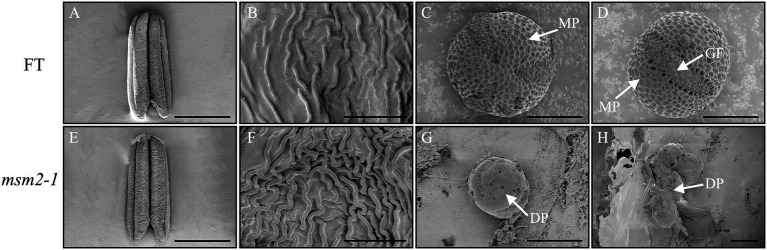
SEM analysis of the anthers and pollen grains in the wild-type “FT” and *msm2-1* mutant. The anthers of the wild-type “FT” **(A)** and *msm2-1* mutant **(E)**. Scale bar, 1 mm. The anthers epidermis of the wild-type “FT” **(B)** and *msm2-1* mutant **(F)**, Scale bar. 5 μm. The surface on the pollen exine of the wild-type “FT” **(C,D)** and *msm2-1* mutant **(G,H)**. *MP* mature pollen grains. *GF* germinal furrow, *DP* degenerated pollen grains. Scale bar, 10 μm.

To investigate the anther and pollen exine morphology, the fresh anthers of the wild-type “FT” and the *msm2-1* were inspected by scanning electron microscopy. The SEM observation of the anther and pollen surface depicted the *msm2-1* mutant had a slightly smaller anther versus the wild type at the anther maturation stage (Figures 3A,E). The cuticle ridges of the anther epidermis in wild-type “FT” are loosely distributed while the cuticles of *msm2-1* are arranged relatively tightly (Figures 3B,F). The mature pollen grains of wild-type “FT” rounded surfaces having a reticular structure and the visible germinal furrow (Figures 3C,D), while pollen grain surface of mutant *msm2-1* is smooth with neither reticular structure nor germination furrow, and finally degraded to pieces (Figures 3G,H).

**Figure 4:**
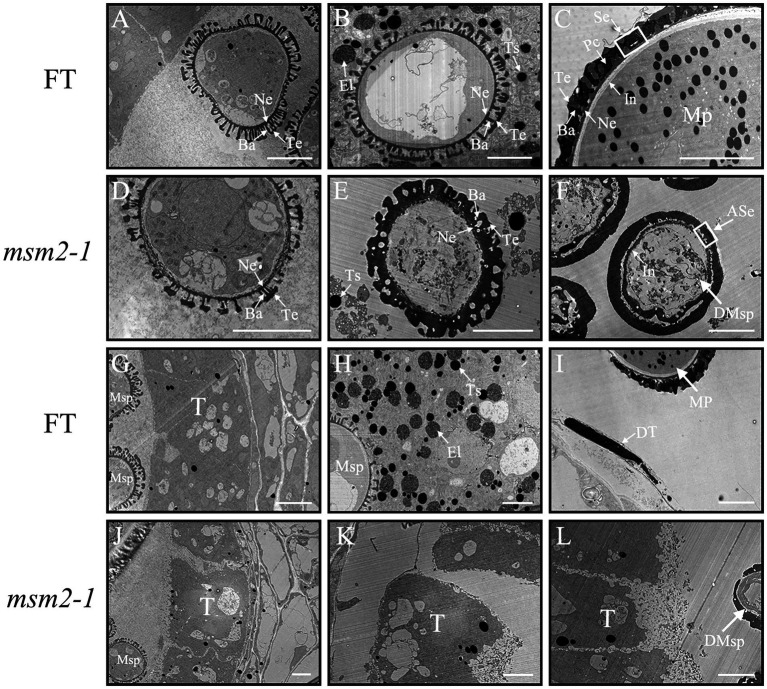
TEM analysis of pollen wall and tapetum development in the wild-type “FT” and *msm2-1* mutant. The process of pollen exine development in wild-type “FT” and *msm2-1* mutant at early (BUDS < 2.0 mm; **A,D**), middle (2.0 mm < BUDS <3.0 mm; **B,E**), late (BUDS > 3.0 mm; **C,F**) stage. The process of tapetum development in wild-type “FT” and *msm2-1* mutant at early (BUDS <2.0 mm; **G,J**), middle (2.0 mm < BUDS <3.0 mm; **H,K**), late (BUDS >3.0 mm; **I,L**) stage; Scale bar, 5 μm. T, Tapetal layer; DT, Degraded tapetum; Msp, Microspores; DMsp, Degenerated microspores; MP, Mature pollen grains, Ba, Bacula; Te, Tectum; Se, Sexine; In, Intine; ASe, Abnormal sexine; El, Elaioplast; Ts, Tapetosome.

To reveal the *msm2-1* pollen grain’s developmental defects in detail, TEM was conducted to observe the anther development. In the early stage of the anther development, there was no distinct difference between the wild-type “FT” and the *msm2-1* mutant (Figures 4A,D,G,J). In the middle stage of the anther development, the tapetum cells of wild-type “FT” began to degrade and secrete lipid droplets and protein substances (Figure 4H). These substances were deposited on the wild-type pollen exine, which formed with the well-organized layers including the tectum, bacula, and foot layer (Figure 4B). The degradation of the tapetum was delayed in *msm2-1* mutant (Figure 4K) and the skeletal structure of the pollen exine was thickened continuously (Figure 4E). At the late stage of the anther development, the pollen coat substances produced by tapetum degradation filled the sexine of the pollen exine to form the pollen coat in the wild-type “FT” (Figures 4C,I). However, the pollen of *msm2-1* was surrounded by a thick abnormal pollen exine structure, while the filling of skeleton structure by pollen coats was not observed in *msm2-1* (Figure 4F). In the wild type, a white pollen intine structure could be seen on the inner side of the pollen wall, while the pollen inner wall structure of the mutant *msm2-1* was vague and amorphous (Figures 4C,F).

### Identification of the causal gene of *msm2-1* by MutMap and KASP

**Figure 5:**
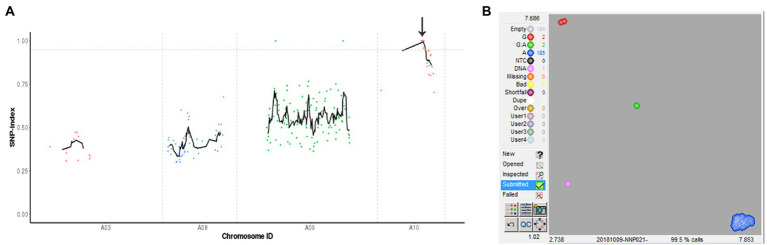
Identification of the *msm2-1/2/3* candidate gene. **(A)** SNP index plot of ten chromosomes was produced by MutMap analysis. The x-axis represents the positions of the ten chromosomes and the y-axis represents the SNP index. The dotted pink line represents the index threshold (0.95). **(B)** The results of SNP genotyping arrays of *BraA10g019050.3C.*

A modified MutMap method was applied to identify the mutant gene. By genome resequencing of the wild-type “FT,” *msm2-1* mutant and the F_2_ mutant DNA pools, 93,975,658, 58,702,874 and 166,730,557 high-quality reads were obtained, respectively. Whereafter, these clean reads were aligned with the reference genome, resulting in 98.00%, 99.00% and 98.83% mapped to it, respectively. With the SNP index of 0.95 as the threshold, we mapped 1.08 Mb (14,034,596–15,116,841) candidate regions on chromosome A10 and detected three SNP mutations (SNP A10, 140,813,37; SNP A10, 143,901,24; SNP A10, 146,585,70), which were located at exons and resulted in nonsynonymous mutations (Figure 5A; Supplementary Table S8).

For further determining the candidate genes, we adopted KASP technology to design primers for three candidate SNPs, and genotyping was performed in “FT,” mutant *msm2-1*, F_1_ and F_2_ mutant plants. The results showed that the genotypes of three SNPs in wild type, mutant and F_1_ generation were G: G, A: A and G: A, respectively. The gene phenotypes of *BraA10g019050.3C* SNP (A10:14,081,337) in F_2_ generation were all A: A (Figure 5B) and the SNPs of the other two genes (A10:14,390,124; A10:14,658,570) appeared six and nine heterozygous types (G: A) in the F_2_ generation sterile plants (Supplementary Figure S2). Therefore, *BraA10g019050.3C* was co-segregated with the mutant phenotype, which further confirmed that *BraA10g019050.3C* was the mutant gene of *msm2*-1. *BraA10g019050.3C* encodes a PHD-finger protein sharing 88% identity with the *Arabidopsis MS1*, which functions as a transcriptional activator that regulates tapetal development and pollen exine biosynthesis. Hence, *BraA10g019050.3C* was named *BrMS1*.

### Sequence analysis of *BrMS1* in *msm2-1/2/3* mutants

**Figure 6:**
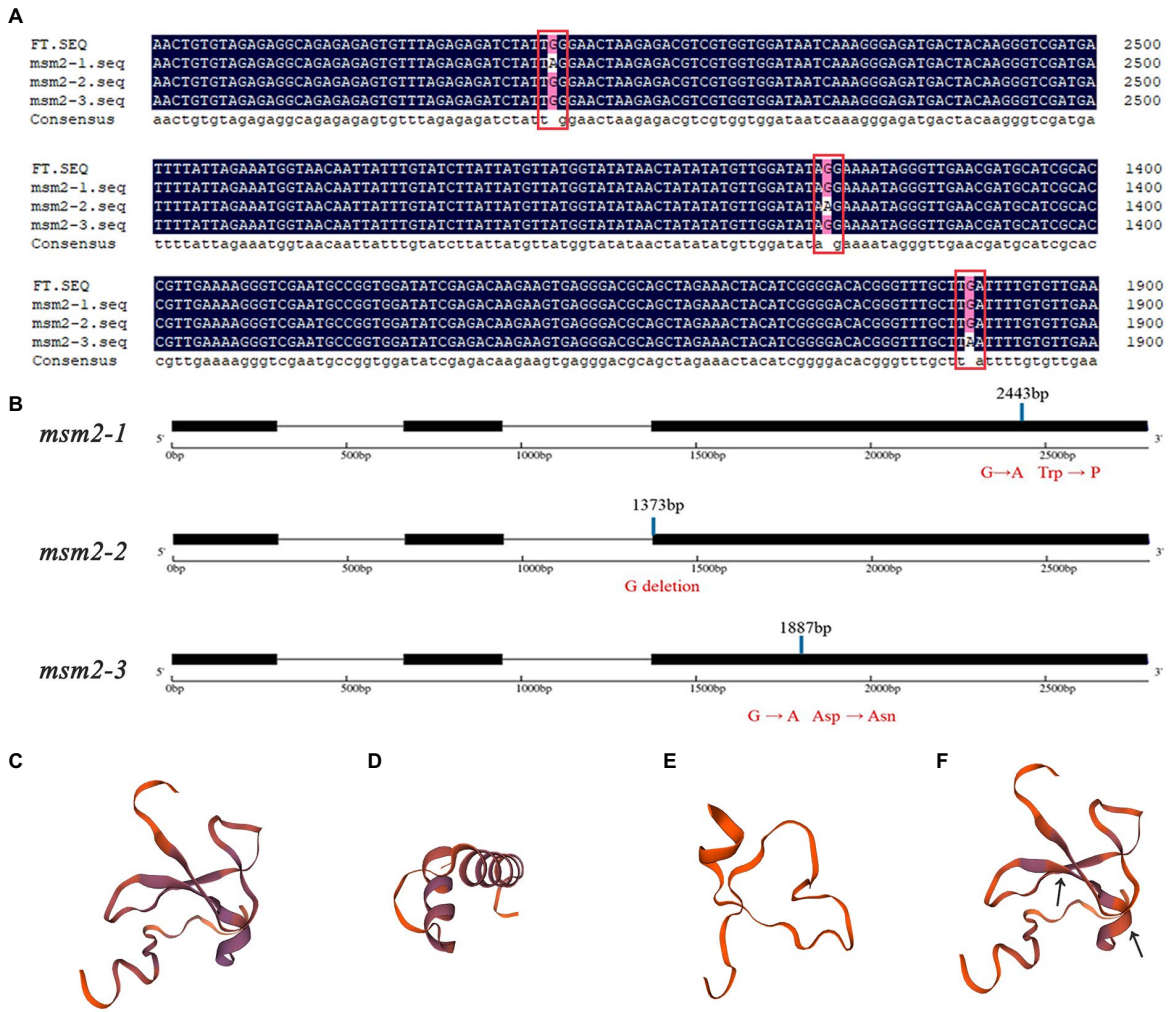
Sequence alignment and structure analysis of *BrMS1* between the wild-type “FT” and *msm2-1/2/3* mutants. Comparison of the *BraA10g019050.3C* nucleotide sequence **(A)** and structure **(B)** between the wild-type “FT” and *msm2-1/2/3* mutants. Three-dimensional protein structure of *BraA10g019050.3C* in the wild-type “FT” **(C)**, *msm2-1* **(D)**, *msm2-2* **(E)** and *msm2-3* **(F)**.

The mutants *msm2-1*, *msm2-2* and *msm2-3* are reciprocal allelic mutants, which means that the function of *BrMS1* can be verified in the other two mutants. The results of the *BraA10g019050.3C* gene cloned from three mutants proved that at 2443 bp in exon 3 of the mutant *msm2-1*, the base sequence changed from G to A and the amino acid changed from tryptophan to terminator codon, leading to the termination of translation; At 1372 bp in the end of the second intron of mutant *msm2-2*, the base sequence changed from G to A, resulting in cutting off by mistake at the first base of third exon and giving rise to frameshift mutation; At 1887 bp in the third exon of *msm2-3*, the base sequence changed from G to A, and the amino acid changed from Asp to Asn (Figures 6A,B). The amino acid transformation attributed to the *BrMS1* mutation likewise contributed to differences in the three-dimensional structure of the protein (Figures 6C–F). These results hinted that the mutation in *BrMS1* led to the male sterile phenotype in *msm2-1/2/3* and verified its function in stamen development.

### Structural and phylogenetic analyses of BrMS1

**Figure 7:**
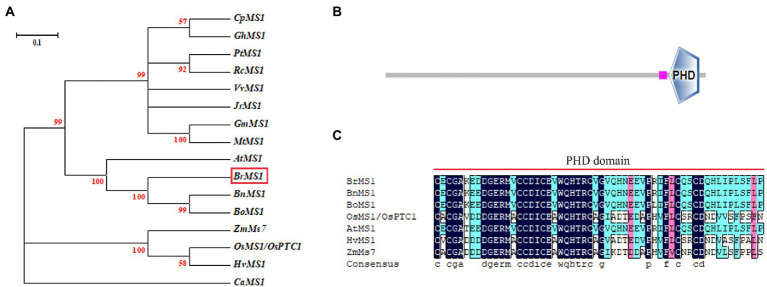
PHD-finger domain sequence alignment in different species and phylogenetic analysis of *MS1*. **(A)** Phylogenetic analysis of MS1 and its related proteins. An unrooted maximum likelihood tree was created by MEGA 3.1 using the MS1-related sequences from *Carica papaya* (*Cp*), *Gossypium hirsutum* (*Gh*), *Populus trichocarpa* (*Pt*), *Ricinus communis* (*Rc*), *Vitis vinifera* (*Vv*), *Juglans regia* (*Jr*), *Glycine max* (*Gm*), *Medicago truncatula* (*Mt*), *Arabidopsis* (*At*), *Brassica rapa* (*Br*), *Brassica napu* (*Bn*), *Brassica oleracea* (*Bo*), *Zea mays* (*Zm*), *rice* (*Os*), *barley* (Hv), *Capsicum annuum* (*Ca*). **(B)** Conserved domain structure of *BrMS1.* **(C)** PHD-finger domain sequence alignment. Residues with 100% similarity are shaded in black. Those with ≥75% similarity are shaded in pink. Those with ≥ 50% similarity are shaded in blue.

To elucidate the evolutionary relationship between BrMS1 and its close homologs, an unrooted tree of BrMS1 and its 16 homologs from moss to flowering plants was constructed (Figure 7A). BrMS1 was clustered with many dicotyledons, and located in the same clade as AtMS1, BnMS1, BoMS1. BrMS1 has a zinc finger protein (PHD-finger) domain (Figure 7B). The PHD-finger domain is functionally conserved in a variety of plants. We compared the sequences of the PHD-finger structural regions among BrMS1 and homologous sequences in *Arabidopsis*, *rice*, *maize*, and *wheat*. The amino acid sequence of the PHD-finger domain sequence in Chinese cabbage was 90% consistent with that of AtMS1 (Figure 7C).

### Spatio-temporal expression pattern of *BrMS1* in *msm2-1/2/3* mutants

**Figure 8:**
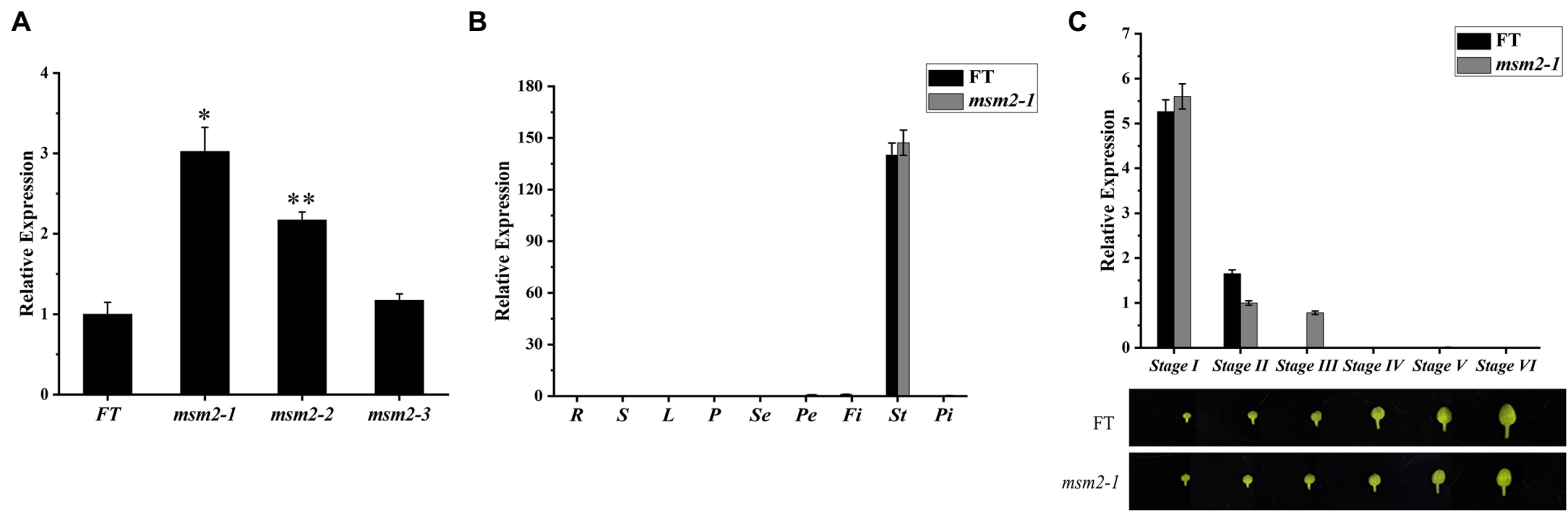
Expression pattern of *BrMS1* between the wild-type “FT” and *msm2-1/2/3* mutants. **(A)** Analysis of *BrMS1* expression in the wild-type “FT” and the allelic mutants *msm2-1/2/3.* Asterisks indicate statistically significant differences determined using a *t*-test (^*^p < 0.05; ^**^p < 0.01). **(B)** Expression of *BrMS1* in the roots (*R*), stems (*S*), leaves (*L*), pods (*P*), sepal (*Se*), petal (*Pe*), filament (*Fi*), stamen (*St*) and pistil (*Pi*) of the wild-type “FT” and *msm2-1* mutant. **(C)** Analysis of *BrMS1* expression in different grades of buds in wild-type “FT” and *msm2-1* mutant. The grading rules for flower buds are the same as those for paraffin sections.

qRT-PCR analysis was conducted to recognize the spatial and temporal expression patterns of *BrMS1* in wild-type “FT” and mutants *msm2-1/2/3*. The expression level of *BrMS1* in the buds of the mutants *msm2-1/2/3* was higher than in the wild-type “FT” line and the expression level of the *BrMS1* gene in the mutant *msm2-1/2* was significantly higher than that of the wild-type “FT,” while the expression level of the *BrMS1* gene in *msm2-3* was slightly higher than that of the wild-type “FT” (Figure 8A). The results were likewise made clear that *BrMS1* was specifically expressed in stamens at stage I and stage II (i.e., the stage of tetrad to uninucleate microspore release). Afterwards, the expression level of *BrMS1* declined rapidly and was almost undetectable in the mature anthers (Figures 8B,C). The expression patterns of *BrMS1* in allelic mutants *msm2-2/3* are same as that in mutant *msm2-1* (Supplementary Figure S3).

### *BrMS1* functions as a transcription factor with transcriptional activation activity

**Figure 9:**
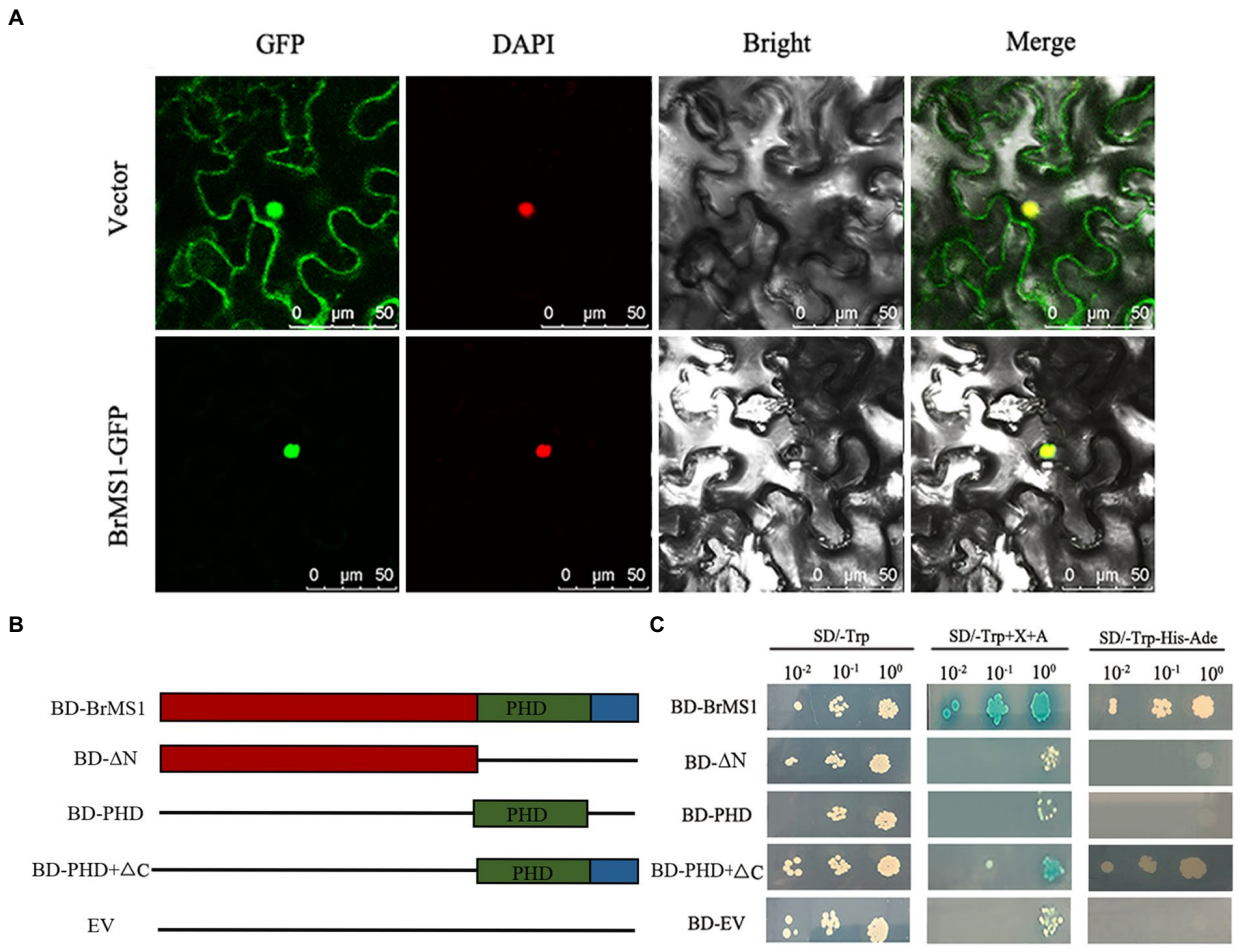
Subcellular localization and transcriptional activation assay of the *BrMS1.* **(A)** Subcellular localization of *BrMS1* in Tobacco leaf. DAPI staining was used as a nuclear marker. Scale bar, 50 μm. **(B)** The schematic diagram displays various truncated constructs and pGBKT7 empty vector used for the transcriptional activation assay. **(C)** Transcriptional activation assay of *BrMS1* in the Y2H yeast strain. BD GAL4–DNA binding domain, EV empty vector. The BD–EV used as a negative control.

Previous studies have shown that *Arabidopsis MS1* functions as a transcriptional activator that regulates pollen and tapetum development (Ito and Shinozaki, 2002). Herein, to verify if *BrMS1* functions as a TF in Chinese cabbage, a subcellular localization assay was initially performed. The transient expression assay showed that the GFP signal was only detected in the protoplast nucleus and indicated that *BrMS1* encodes a PHD-finger protein localized in the nucleus, supporting that it functions as a TF (Figure 9A).

For analyzing whether *BrMS1* exhibits transcriptional activity activation, the full-length CDS and three truncated fragments of *BrMS1* (ΔN, ΔC, PHD) were ligated into a pGBKT7 vector to generate BD-*BrMS1*, BD-ΔN, BD-ΔC, and BD-PHD fusion constructs (Figure 9B). The results showed all of vectors transformed yeast cells could grow in the control medium (SD/-Trp), but only BD-*BrMS1* and BD-ΔC could grow in the selective medium (SD/-Trp/-His/-Ade). The findings demonstrated that *BrMS1* exhibited transcriptional activation activity in yeast (Figure 9C). Subsequently, the characteristics of the deleted fragments were extensively compared. We discovered that a length of 12 C-terminal amino acid residues were required to activate activity. Taken together, these results suggested that *BrMS1* was a nuclear-localized TF with a length of 12 C-terminal amino acid transcriptional activation activity.

### *BrMS1* affects the transcription level of genes associated with tapetal PCD and pollen wall development

**Figure 10:**
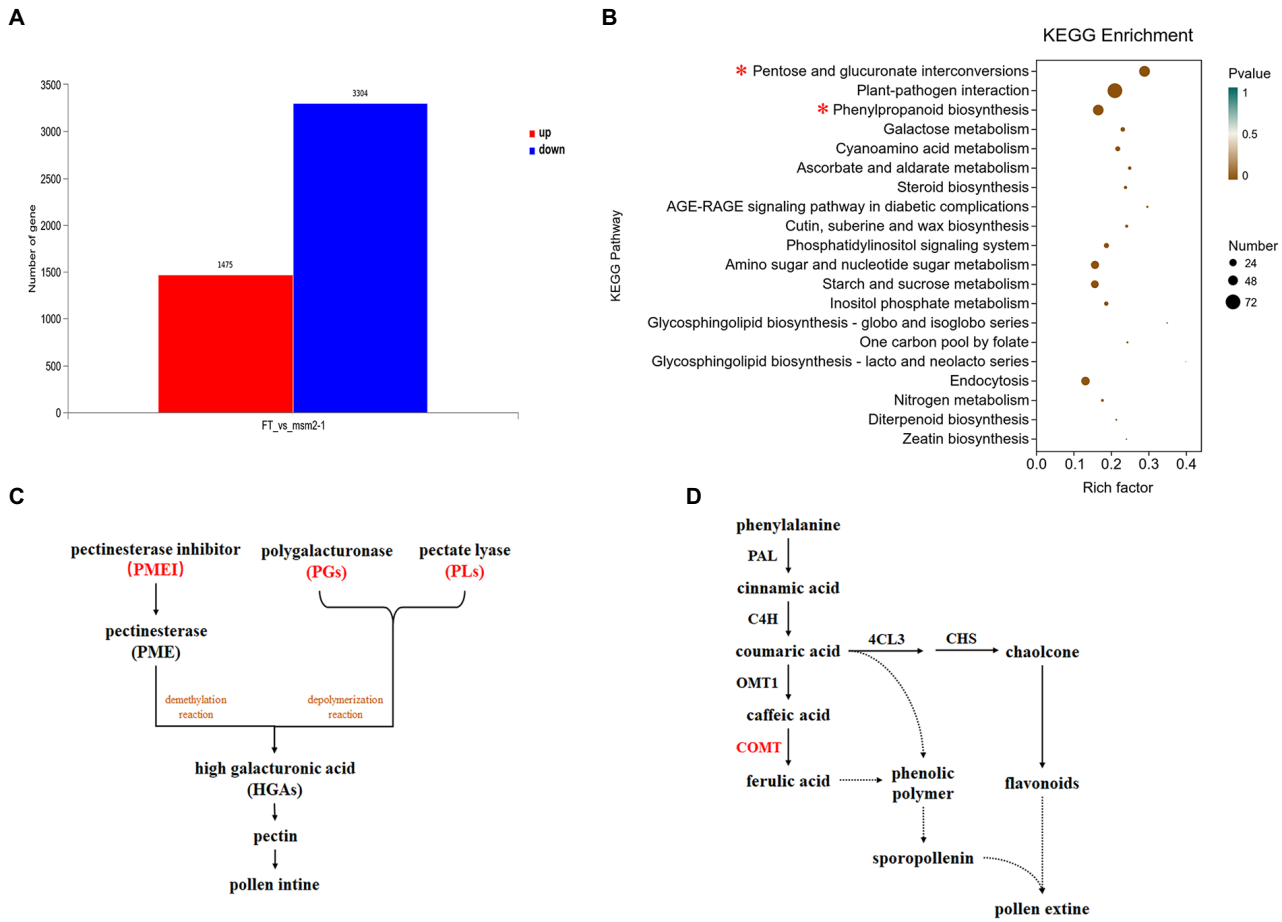
RNA-seq analysis between the wild-type “FT” and *msm2-1* mutant. **(A)** The number of different expression genes (DEGs) in the anthers between the wild-type “FT” and *msm2-1* mutant. **(B)** Kyoto Encyclopedia of Genes and Genomes enrichment results of the DEGs. **(C)** Schematic of pentose and glucuronate interconversions pathway related to pollen development. The red part represents the key enzymes enriched by the differentially expressed genes. **(D)** Schematic of phenylpropanoid metabolism pathway related to pollen development. The red part represents the key enzymes enriched by the differentially expressed genes.

As a TF, *BrMS1* is expected to affect the anther development by regulating the expression of downstream genes. To identify genes that might be regulated by *BrMS1*, we performed a comparative transcriptome analysis of anther in wild-type “FT” and the *msm2-1* using RNA-Seq. 4,779 DEGs were identified between the wild-type “FT” and the *msm2-1*, involving 1,475 upregulated genes and 3,304 downregulated genes in *msm2-1* (fold change ≥ 10, false discovery rate, FDR < 0.01; Figure 10A). The KEGG analysis displayed that DEGs were enriched in multiple metabolic pathways associated with pollen development, including the pentose and glucuronate interconversions pathway (53 DEGs), phenylpropanoid metabolism pathway (52 DEGs), galactose metabolism pathway (21 DEGs) as well as cutin, suberine and wax biosynthesis pathway (15 DEGs; Figure 10B). In the pentose and glucuronate interconversions pathway, several genes related to pectin biosynthesis which affected the formation of pollen intines were identified, containing 10 pectinesterase inhibitor genes (*PMEIs*), 13 polygalacturonases genes (*PGs*) and 11 pectate lyasegenes (*PLs*; Figure 10C; Supplementary Table S9). Most of them were down-regulated in *msm2-1* and some of these genes expressed specially in the wild-type “FT” such as *PMEIs* (*BraA06g033760.3C* and *BraA08g011580.3C*), *PGs* (*BraA02g039370*.*3C*, *BraA02g039400.3C* and *BraA09g065620.3C*), *PLs* (*BraA09g059390.3C* and *BraA06g039690.3C*). The phenylpropanoid pathway splits into two main branches leading to hydroxycinnamic acids and flavonoids, both of which are related to the synthesis of pollen exine (Preston et al., 2004). The expression levels of some genes involved in the synthesis of key enzymes e.g., cinnamyl alcohol dehydrogenase, feruloyl-CoA, caffeoyl-CoA were significantly changed in the mutant *msm2-1*, among which *COMT* (*BraA02g024180.3C*) was specifically expressed in the wild-type “FT” (Figure 10D; Supplementary Table S10). RNA-seq profiling suggested that the defect in *BrMS1* affected the transcript levels of genes involved in pollen intine and exine biosynthesis metabolic pathways in *msm2-1*.

In order to assess whether the defect in *BrMS1* broadly affects other pollen development pathways, we compared gene expression associated with pollen development between the wild-type “FT” and the *msm2-1*. In *Arabidopsis*, *AtMS1* regulates the expression of *PCPs* and *PCLs* genes, which participate in pollen coat formation (Lu et al., 2020; Zhang et al., 2021). In our study, several *PCPs* genes such as *GRPs* and *EXLs* exhibited a prominent reduction in the mutant *msm2-1*, but no significant changes were observed in the expression of *PCL* synthesis genes such as *KCS7/5/11/21* (Supplementary Table S11). Excessive deposition of sporopollenin on the pollen primexine of the *msm2-1* was observed by TEM. The results of the RNA-seq also showed that some crucial sporopollenin synthesis genes (*SSGs*) such as *ACOS5*, *MS2*, *CYP703A2*, *PKSA* were up-regulated in the *msm2-1* (Supplementary Table S12). In the tapetum development regulatory network, *MS188* is the upstream regulatory factor of *MS1*. The expression of *MS188* genes (*BraA10g013970.3C* and *BraA02g013010.3C*) in the mutant *msm2-1*, was higher than in the wild-type “FT.” *AMS* (*BraA07g004220.3C* and *BraA03g043400.3C*), which was the upstream gene of *MS188*, also up-regulated expression in *msm2-1* (Supplementary Table S13). The degradation of tapetum cells is regarded as a process of PCD, and papain-like cysteine proteases play a key control role in the timely degradation of tapetum cells (Liu et al., 2018; Wang et al., 2021). *CEP1* is a crucial executor during tapetal PCD and that proper *CEP1* expression is necessary for timely degeneration of tapetal cells and functional pollen formation in *Arabidopsis thaliana* (Zhang et al., 2014). *BraA03g026210.3C*, a homologous gene of *CEP1*, was up-regulated in *msm2-1*. We also observed the downregulation of the other two cysteine proteases genes *CP1* (*BraA10g004970.3C* and *BraA08g033980.3C*) in *msm2-1* compared with the wild-type “FT” (Supplementary Table S13). Overall, these findings suggested that *BrMS1* might directly or indirectly regulate the expression levels of *PCPs*, *SSGs* and *Cys* proteases genes, further resulting in pollen abortion in *msm2-1*.

**Figure 11:**
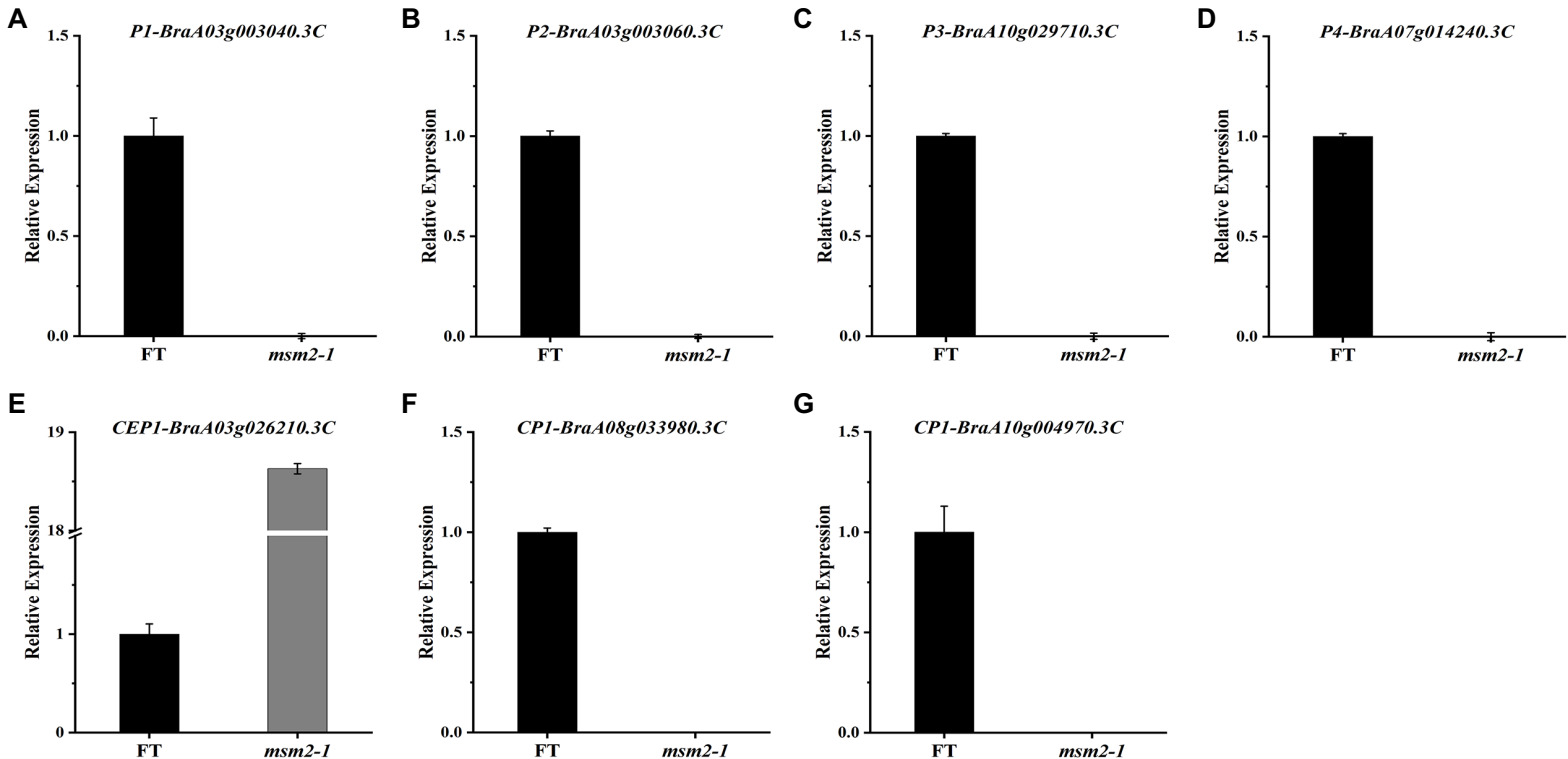
The quantitative reverse transcription polymerase chain reaction analysis of DEGs between the wild-type “FT” and *msm2-1* mutant. **(A–D)** The expression level of *pollen coat proteins* in anthers between the wild-type “FT” and *msm2-1* mutant. **(E–G)** The expression level of papain-like cysteine proteins in anthers between the wild-type “FT” and *msm2-1* mutant.

To test the reliability of RNA-seq data, we verified the transcript levels of four PCP genes and three cysteine protease genes between the wild-type “FT” and the mutant *msm2-1* by qRT-PCR, and the analyses showed that their mRNA levels were all significantly reduced in *msm2-1*, apart from *CEP1*, which was up-regulated (Figure 11). These findings are consistent with the results from the RNA-seq analyses.

## Discussion

The application of male sterility lines is an effective approach to commercial hybrid seed production and heterosis utilization in Chinese cabbage. Moreover, male sterility mutant is an important material to study pollen development and nuclear-cytoplasmic interactions. The creation of male sterile materials through artificial mutation has become a crucial mean of male sterility research. Discovery and research on male sterile mutants also had been a hot topic in research in various species. In this study, we cloned firstly the male sterility gene *BrMS1* in Chinese cabbage by forwarding genetics strategy and revealed the critical role of *BrMS1* in pollen development. In addition, the male sterile mutants we discovered have the characteristics of complete abortion, genetical stability and no concomitant adverse traits, which can be adopted as excellent sources of male sterility for Chinese cabbage hybrid seed production.

### The defect of *BrMS1* caused tapetum cell delayed degeneration and abnormal pollen wall in *msm2-1/2/3* mutants

Tapetum timely degradation is essential for the pollen development in plants. The forms of abnormal tapetal development led to male sterile are diverse (Sanders et al., 1999; Zhang et al., 2011; Zhu et al., 2011; Zhang and Yang, 2014). Premature tapetum cell death caused the male sterile in rice *osmyb80* and *bm1* mutants (Pan et al., 2020; Xiang et al., 2021). Loss of *RGAT1* function also induced the premature degeneration of tapetal cells and pollen abortion in *Arabidopsis* (Qian et al., 2021). In addition, delayed tapetum degradation brought out defective mature pollen in rice *osmyb103* and *sts1* mutants (Lei et al., 2022; Yuan et al., 2022). In the present study, delayed tapetum degeneration is a hallmark defect in *msm2-1/2/3*, but the tapetum cells enlargement was not observed in *msm2-1/2/3* (Figures 2M–X). This phenotype was distinct from other tapetal PCD delayed mutants that have been described, such as *gamyb* and *tdr* mutants, which exhibited tapetal defects involving tapetum cell enlargement (Kaneko et al., 2004; Li et al., 2006), These findings suggested that the *BrMS1* mutation in *msm-1/2/3* mutants induced the delayed degradation of tapetum, but did not affect the morphology of tapetum cells.

Known tapetal PCD mutants also showed different types of pollen exine patterning. The manifestations of pollen exine abnormal in *msm2-1* mutant were different from other *MS1* homologous mutants. In the *Arabidopsis ms1* mutant, exine formation is abnormal, with initial primexine deposition but with only limited sporopollenin deposition (Ito and Shinozaki, 2002; Ariizumi et al., 2003; Vizcay-Barrena and Wilson, 2006). In the *rice*, the *osms1*/*ptc1* exines exhibited a two-layer structure, the sexine was abnormal because of the missing or decrease bacula (Yang et al., 2019b). In the *maize*, ubisch bodies were not enlarged in mutant *ms7-6007* and exine was much thinner than WT (An et al., 2020). In this study, the *msm2-1* mutant could form a complete pollen exine structure in the early stage of pollen development yet not observed the *PCP*s filled the pollen exine at the late stage. The excessive deposition of sporopollenin resulted in the pollen exine structure continuously thickened and formed a doughnut-like structure in the late stage of pollen development (Figures 4B,C,E,F). The SEM observation of pollen also exhibited a lack of elaborate patterning, giving rise to a smooth surface in *msm2-1* mutant (Figures 3C,D,G,H). In addition, abnormal morphology of pollen inner wall was observed in *msm2-1*. Taken together, loss of function of *BrMS1* results in delayed tapetal degeneration and pollen wall abnormal formation, and these developmental defects ultimately cause complete male sterility in the Chinese cabbage.

### *BrMS1* as a PHD-finger transcriptional activator negatively regulates its own expression

To decipher the molecular mechanism of the causal gene, MutMap and KASP analyses revealed that *BrMS1* (*BraA10g019050.3C*) encoded a PHD-finger transcriptional factor, was the causal gene in mutant *msm2-1* and confirmed function by *BrMS1* cloning in the allelic mutants *msm2-2/3* (Figures 5, 6). A previous study has shown that the PHD-finger motif was required for *MS1* function in *Arabidopsis* (Ito et al., 2007). The mutation of lacking the PHD-finger motif in both *OsMS1* and *PTC1*, caused male sterility in rice (Ito et al., 2007; Li et al., 2011; Yang et al., 2019b). Here, the base variations from G to A at 2443th of *msm2-1*, 1373th of *msm2-2* and 1887th of *msm2-3* made the PHD-finger domain abnormal in the allelic mutants and eventually led to pollen abortion (Figures 6A,B). Therefore, this demonstrates to some extent that the PHD-finger motif is indispensable for the development of anthers in Chinese cabbage.

Previous studies have implied *MS1/PTC1* could down-regulate their own expression in wild-type plants, and conjectured that *MS1* down-regulated its expression may due to conformational problem with the truncated mutant polypeptides and/or that the PHD-finger motif binding to its own promoter (Yang et al., 2007; Li et al., 2011). In this paper, qRT-PCR analysis revealed that *BrMS1* transcripts were increased to varying degrees in the *msm2-1/2/3* mutant, implying that self-regulatory based upon a normal gene functional could limit the expression of *BrMS1* in Chinese cabbage (Figure 8A). The expression of *BrMS1* in *msm2-1/2* allele mutants showed a greater expression compared with in *msm2-3*, we speculated that this was on account of the smaller difference in the 3D structure of the *BrMS1* gene in *msm2-3* versus the wild type “FT” (Figures 6C–F).

### *BrMS1* plays a key role in Chinese cabbage pollen development

To gain a better understanding of how *BrMS1* affects pollen development, we produced transcriptomes of the wild-type “FT” and the *msm2-1* mutant to identify potential genes and pathways involved in pollen development. The pollen coat rich in lipids and proteins plays an important role in the attachment and recognition of the pollen to the stigma (Kobayashi et al., 2021). The pollen coat is necessary for fertility, as pollen coat mutants in rice lead to the humidity-sensitive male sterility (Xue et al., 2018; Yu et al., 2019; Chen et al., 2020). In this study, RNA-seq profiling demonstrated that pollen coat proteins *GRPs* and *EXLs* significantly down-regulated in *msm2-1* compared with the wild-type “FT” (Supplementary Table S11). Accumulating reports implicated that papain-like cysteine proteases play a role in tapetal PCD, our results indicated that cysteine proteases *BrCEP1* (*BraA03g026210.3C*) was up-regulated and the other two cysteine proteases genes *BrCP1* (*BraA10g004970.3C* and *BraA08g033980.3C*) were down-regulated in *msm2-1* (Supplementary Table S13). These findings, to some extent, revealed a potential cause of the delayed degradation of tapetum in the *msm2-1* mutant. KEGG pathway analysis indicated that differentially expressed genes were significantly enriched in the pentose and glucuronate interconversions pathway. In this pathway, most DEGs involved in pectin biosynthesis, the main component of the pollen intine. *HGAs* are the most abundant polymer of pectin (Wolf et al., 2009). The HGA-modifying process can be strictly regulated by multiple cell wall enzymes. It has been demonstrated that the demethylesterification of *HGAs* could be spatiotemporally controlled by pectin methylesterases (*PMEs*) and PMEs activity might be regulated by a specific proteinaceous *PME* inhibitors (*PMEIs*; Camardella et al., 2000). Polygalacturonases (*PGs*) and pectin lyases (*PLs*) can depolymerize *HGAs* (Wakabayashi et al., 2003). Most of *PMEIs*, *PGs* and *PLs* were down-regulated in *msm2-1* (Figure 10C; Supplementary Table S9). Thus, we speculated that the defection of *BrMS1* regulated the development of pollen intine by affecting the expression of genes relevant to pectin biosynthesis in *msm2-1* mutant. However, the specific way in which *BrMS1* regulates these fertility-related genes will require further investigation.

**Figure 12:**
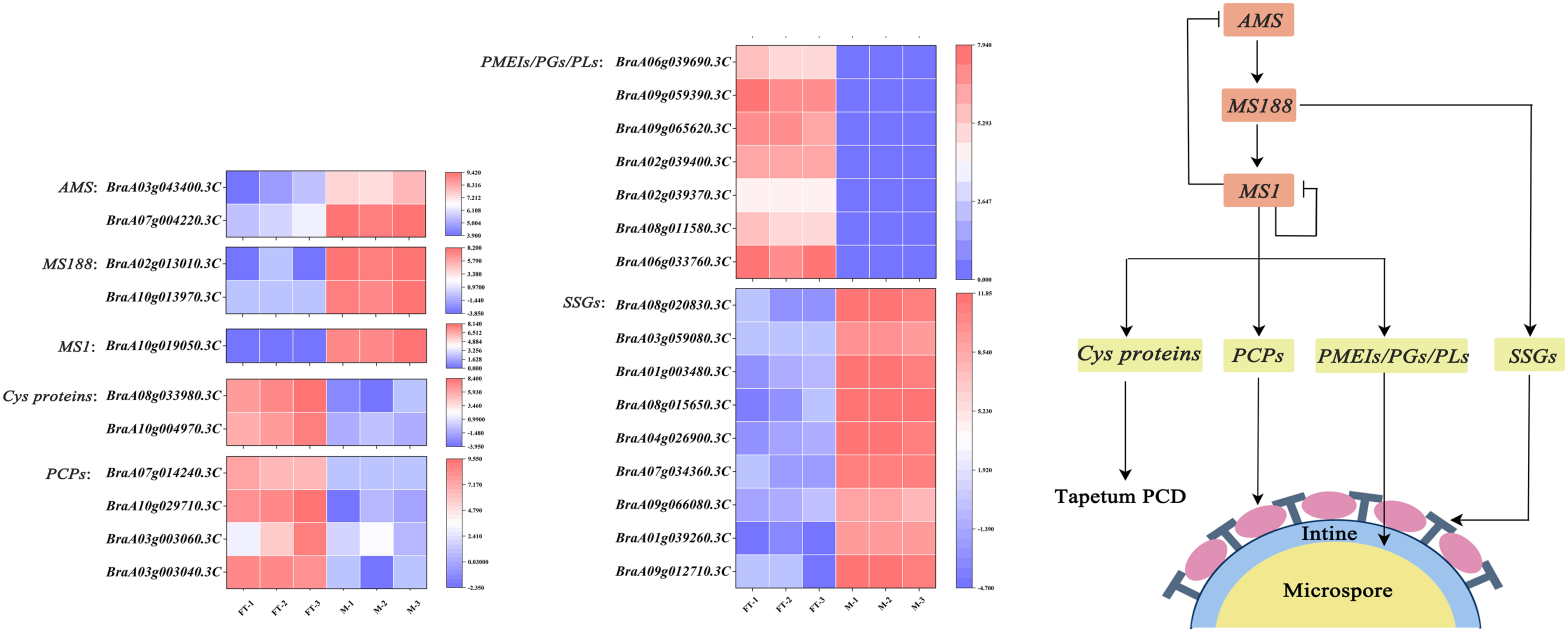
Proposed working model for *BrMS1* regulation of male fertility in Chinese cabbage. *MS1* which encodes a PHD-finger transcription factor could regulate the tapetum programmed cell death by influencing the transcription level of *Cys* proteins. *MS1* functions in pollen coats and pollen intine formation by mediating the expression of related genes in anthers. In addition, *MS1* could negatively regulate the transcription level of itself and the upstream regulator *AMS*, which in turn affects the expression of *SSGs* to control the formation of pollen exine. *MS1* plays an important role as a downstream regulator in the male fertility regulatory network.

Sporopollenin is the main component of the pollen exine and that its biosynthesis is closely related to fatty acid and phenylpropanoid metabolism (Liu and Fan, 2013; Shi et al., 2015). In this study, DEGs was likewise enriched in the phenylpropanoid metabolism pathway. The expression of some key enzymes in this pathway were significantly changed in *msm2-1* (Figure 10D; Supplementary Table S10). In addition, several *SSGs* were up-regulated expression in mutant *msm2-1*. This finding was consistent with the excessive deposition of sporopollenin of the *msm2-1* genotype (Figure 4; Supplementary Table S12). As we know that *MS188* directly regulates sporopollenin biosynthesis genes, and *MS188* is an upstream regulator of *MS1* in the tapetum regulatory network (*DYT1-TDF1-AMS-MS188-MS1*; Xiong et al., 2016; Wang et al., 2018). Herein, the expression level of *MS188* was up-regulated (Supplementary Table S13). In addition, several reports have shown that *MS1* could negatively regulate *AMS* expression, possibly through chromatin remodeling, or indirect protein degradation (Ferguson et al., 2017). This complex network of feedback loops is critical for the anther development. We suspected that mutation in *BrMS1* broke the balance of this negative feedback mechanism, resulting in the up-regulation expression of *AMS* and its downstream target gene *MS188*. While the *SSGs* directly regulated by the *MS188* were also up-regulated, giving rise to pollen exine abnormal in the mutant *msm2-1*. *BrMS1* as a TF could not only regulate the expression of downstream potential pollen development-related genes, but also effect on the expression of upstream genes (Figure 12). Hence, *BrMS1* plays an important role in maintaining the stable and timely expression of various TFs in the tapetum regulatory network.

## Data availability statement

The original contributions presented in the study are publicly available. This data can be found at: https://www.ncbi.nlm.nih.gov/sra//PRJNA856064.

## Author contributions

Data analysis and writing of the manuscript were performed by SD. Study conception and design were performed by SH and HF. JZ, YZ, FS, and GS carried out the experiments. BF took charge of English language editing. All authors contributed to the article and approved the submitted version.

## Funding

The research was supported by the National Natural Science Foundation of China (grant no. 31730082).

## Conflict of interest

The authors declare that the research was conducted in the absence of any commercial or financial relationships that could be construed as a potential conflict of interest.

## Publisher’s note

All claims expressed in this article are solely those of the authors and do not necessarily represent those of their affiliated organizations, or those of the publisher, the editors and the reviewers. Any product that may be evaluated in this article, or claim that may be made by its manufacturer, is not guaranteed or endorsed by the publisher.

## Footnotes

^1^ http://www.ncbi.nlm.nih.gov/BLAST

^2^ https://www.ncbi.nlm.nih.gov/Structure/cdd/wrpsb.cgi

^3^ http://www.geneontology.org/
