# Supplementary material for: Defect in BrMS1, a PHD-finger transcription factor, induces male sterility in ethyl methane sulfonate-mutagenized Chinese cabbage (Brassica rapa L. ssp. pekinensis)
Source: Front Plant Sci. 2022 Aug 18;13:992391. doi: 10.3389/fpls.2022.992391 (PMC9433997; doi:10.3389/fpls.2022.992391)
Supplement: Supplementary file 1 [file Data_Sheet_1.docx]

**
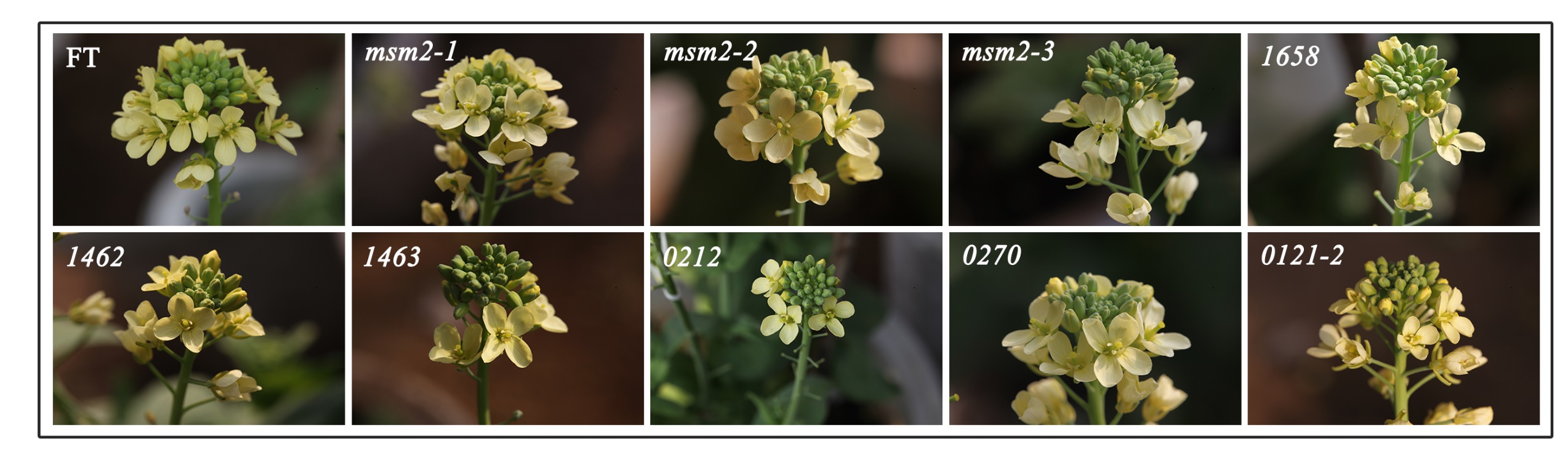
**

**FIGURE S1** The inflorescence of nine genetically stable male sterile mutants and the wild-type ‘FT’

**
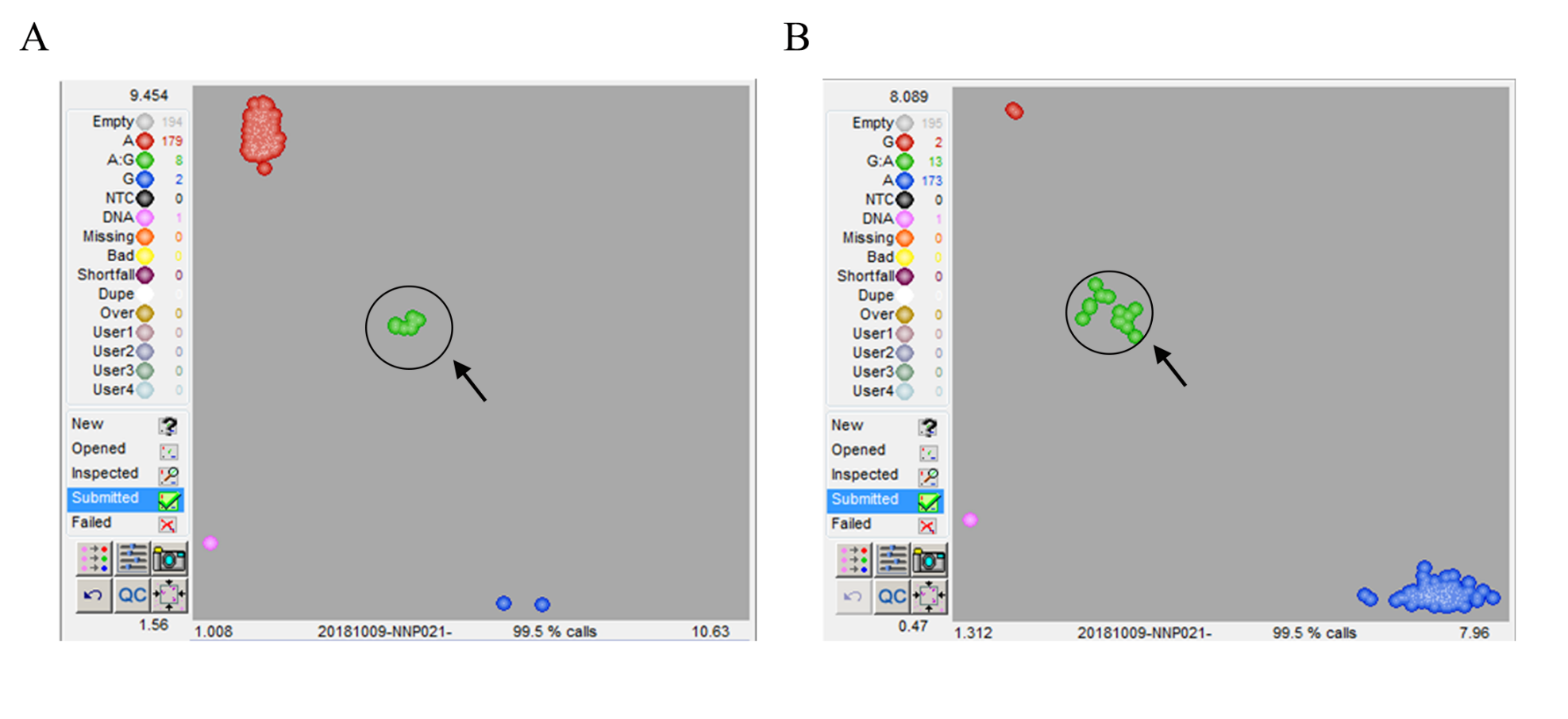
FIGURE S2** The results of SNP genotyping arrays of *BraA10g019600.3C* **(A)** and *BraA10g020140.3C* **(B)**

**
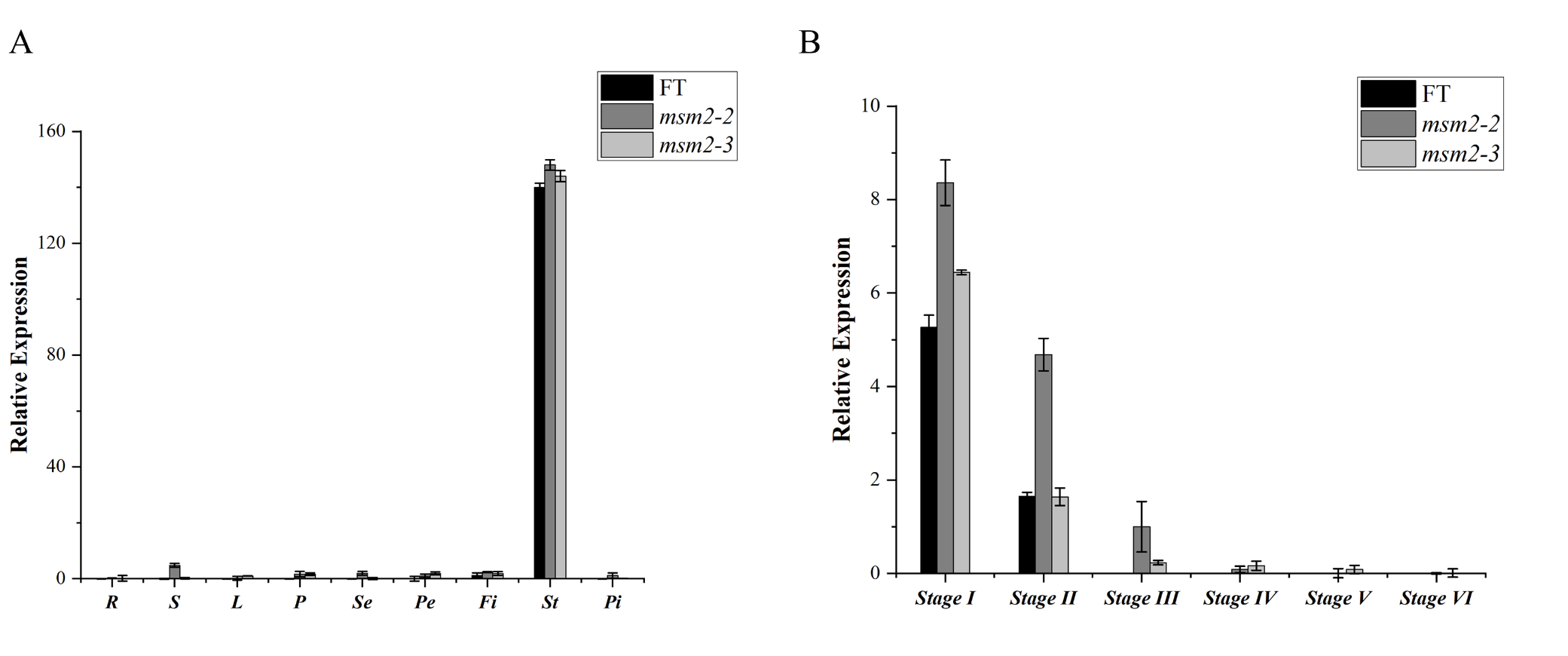
**

**FIGURE S3** **Expression pattern of *BrMS1* between the wild type ‘FT’ and allelic mutant *msm2-2/3*. (A)** Expression of *BrMS1* in the roots (*R*), stems (*S*), leaves (*L*), pods (*P*), sepal (*Se*), petal (*Pe*), filament (*Fi*), stamen (*St*) and pistil (*Pi*) of the wild-type ‘FT’ and mutant msm2-1. **(B)** The expression levels of *BrMS1* in different grades of buds in wild-type ‘FT’ and mutant *msm2-2/3*. (The grading rules for flower buds are the same as those for paraffin sections.)
