# Supplementary material for: Defect in BrMS1, a PHD-finger transcription factor, induces male sterility in ethyl methane sulfonate-mutagenized Chinese cabbage (Brassica rapa L. ssp. pekinensis)
Source: Front Plant Sci. 2022 Aug 18;13:992391. doi: 10.3389/fpls.2022.992391 (PMC9433997; doi:10.3389/fpls.2022.992391)
Supplement: Supplementary file 2 [file Data_Sheet_2.docx]

**Table S1** The allelic test results of nine genetically stable male sterile mutants

|  | **S** | | | | | | | | | |
| --- | --- | --- | --- | --- | --- | --- | --- | --- | --- | --- |
| **M** |  | *0212* | *1462* | *1658* | *msm2-2* | *1463* | *0121-2* | *msm2-1* | *0270* | *msm2-3* |
|  | *0212* |  |  |  |  |  |  |  |  |  |
|  | *1462* | F |  |  |  |  |  |  |  |  |
|  | *1658* | F | F:S=25:25 |  |  |  |  |  |  |  |
|  | *msm2-2* | F | F | F |  |  |  |  |  |  |
|  | *1463* | F | F:S=21:26 | F:S=24:26 | F |  |  |  |  |  |
|  | *0121-2* | F | F | F | F | F |  |  |  |  |
|  | *msm2-1* | F | F | F | F:S=23:27 | F | F |  |  |  |
|  | *0270* | F | F | F | F | F | F | F |  |  |
|  | *msm2-3* | F | F | F | F:S=25:24 | F | F | F:S=26:24 | F |  |

**Note：**The fertile and sterile plants in AB lines of the nine male sterile materials were performed the allelic test by mutual cross. The materials whose segregation ratio of the hybrid progeny conformed to 1:1 were allelic mutants of each other.

**Table S2** Primer sequences used in KASP analyses

| ID | Primer_AlleleF1 | Primer_AlleleF2 | Primer_R |
| --- | --- | --- | --- |
| *BraA10g019050.3C* | GAAGGTCGGAGTCAACGGATTCACCACGACGTCTCTTAGTTCC | GAAGGTGACCAAGTTCATGCTCCACCACGACGTCTCTTAGTTCT | AGATTGGGATTTGGTAGGGAGATACC |
| *BraA10g019600.3C* | GAAGGTCGGAGTCAACGGATTTGAATCAGAGCTAAGGTTAGGGTTAA | GAAGGTGACCAAGTTCATGCTTGAATCAGAGCTAAGGTTAGGGTTAG | GAAACTAGCTTTACTCAATTCCCCTT |
| *BraA10g020140.3C* | GAAGGTCGGAGTCAACGGATTGCAACTAAAGCCATGCATTGAC | GAAGGTGACCAAGTTCATGCTGCAACTAAAGCCATGCATTGAT | ATACCAATACCACTGGCAATCTTCT |

**Table S3** Primers for the genomic sequence of *BraA10g019050.3C*

| Primer name | Primer Sequences (5′–3′) | Length of PCR products (bp) | Tm (°C) |
| --- | --- | --- | --- |
| *BraA10g019050.3C* -1F | ATGTCGAATCTGATTCGAAC | 837 | 58 |
| *BraA10g019050.3C* -1R | ACGGCTTTTTCCACTCCA |  |  |
| *BraA10g019050.3C*-2F | ATCCCCTCAAAGGAAACAATG | 989 | 56 |
| *BraA10g019050.3C* -2R | CATTCGCGTCTTCCAAGAGA |  |  |
| *BraA10g019050.3C* -3F | AAGGCACTTGAGTCGGTCC | 1128 | 57 |
| *BraA10g019050.3C* -3R | ATGTGGAGCGAAGGAGGAT |  |  |
| *BraA10g019050.3C*-4F | AAAGGGAGATGACTACAAGGG | 320 | 57 |
| *BraA10g019050.3C* -4R | AAGGCAGAAAAGAGAGAGGGATAA |  |  |

**Table S4** Primers for the CDS sequence of *BraA10g019050.3C*

| Primer name | Primer Sequences (5′–3′) | Length of PCR products (bp) | Tm (°C) |
| --- | --- | --- | --- |
| *BraA10g019050.3C* -1F | atgtcgaatctgattcgaac | 827 | 56 |
| *BraA10g019050.3C* -1R | TGATACCTTGACAAGAGAATTGGA |  |  |
| *BraA10g019050.3C*-2F | AACTGTAGATGGTCTCAAAAACGG | 1047 | 58 |
| *BraA10g019050.3C* -2R | AAGGCAGAAAAGAGAGAGGGATAA |  |  |

**Table S5** Sequences of the primers used for qRT-PCR

| **Primer name** | **Primer Sequence (5'-3')** |
| --- | --- |
| Actin-F | ATCTACGAGGGTTATGCT |
| Actin-R | CCACTGAGGACGATGTTT |
| *BrMS1*-F  *BrMS1*-R | TGAAAAAGGTGCTGGAGTATTGCT  TTGTTGTGTCCGTTCGTGGC |
| *BrP1*-F | TCACTCCGAAGAGGGCAAAA |
| *BrP1*-R | GTATCCACGCCGCTTTTCAT |
| *BrP2*-F | ATGTTTGAGATTATTCAGGCGG |
| *BrP2*-R | GCTATAGTTGCTGGCACGAGA |
| *BrP3*-F | ACGCCGCTTTTTATTCTATTCAGTC |
| *BrP3*-R | CGTTCCTTTCCCCTTCTTCCTAA |
| *BrP4*-F | AAACCTACCACGGATTTAGAGCG |
| *BrP4*-R | CATGGATGCAAAAGCGACTATTC |
| *BrCP1-* *BraA10g004970.3C* -F | GAAGGTGCTGTGACTCCTGTTAGA |
| *BrCP1-* *BraA10g004970.3C* -R | GTCAGTAATCCACCGCTACATCC |
| *BrCP1-* *BraA08g033980.3C* -F | GAAGGTGCTGTGACTCCCATTAG  TCGCAATCTATGAGTTGTTGTTCTG |
| *BrCP1-* *BraA08g033980.3C* -R |  |
| *BrCEP1*-F | GTCTGATGGACCTTGCTTTTGAGT |
| *BrCEP1*-R | TCGTTGTTCCATAACCTACTGCTG |

**Table S6** Sequences of the primers used for the subcellular location

| Primer name | Primer Sequences (5′–3′) |
| --- | --- |
| *35S: BrMS1*-GFP-F | cagtCACCTGCaaaacaacatgtcgaatctgattcgaac |
| *35S: BrMS1*-GFP-R | cagtCACCTGCaaaatacaaggcagaaaagagagaggga |

**Table S7** Sequences of the primers used for the transcriptional activation assay (The restriction sites are F: *NdeI* and R: *BamHI*.)

| Primer name | Primer Sequences (5′–3′) |
| --- | --- |
| BD-*BrMS1*-F | AGGAGGACCTGCATATGATGTCGAATCTGATTCGAACAGAT |
| BD-*BrMS1*-R | GCAGGTCGACGGATCCAGGCAGAAAAGAGAGAGGGATAAG |
| BD-*BrMS1*-ΔN-F | AGGAGGACCTGCATATGATGTCGAATCTGATTCGAACAGA |
| BD-*BrMS1*-ΔN-R | GCAGGTCGACGGATCCGATCCTCTTATCCTTCTTCTTACTATCTT |
| BD-*BrMS1*-ΔC-F | AGGAGGACCTGCATATGGAGTGTGAATGTGGAGCGAAG |
| BD-*BrMS1*-ΔC-R | GCAGGTCGACGGATCCAGGCAGAAAAGAGAGAGGGATAA |
| BD-PHD-F | AGGAGGACCTGCATATGGAGTGTGAATGTGGAGCGAAGG |
| BD-PHD-R | GCAGGTCGACGGATCCACAGCTTTGACAAAGAAAAATGC |

**Table S8** List of three non-synonymous SNPs

| Chr | Pos | Ref | WT | Mut | SNP index | Location | Gene ID | Annotation |
| --- | --- | --- | --- | --- | --- | --- | --- | --- |
| A10 | 14081337 | G | G | A | 1 | exonic | *BraA10g019050.3C* | PREDICTED: PHD finger protein MALE STERILITY 1 isoform X1 |
| A10 | 14390124 | G | G | A | 1 | exonic | *BraA10g019600.3C* | PREDICTED: somatic embryogenesis receptor kinase 2-like |
| A10 | 14658570 | G | G | A | 1 | exonic | *BraA10g020140.3C* | PREDICTED: LRR receptor-like serine/threonine-protein kinase EFR |

**Table S9** The different expression genes (DEGs) in the pentose and glucuronate interconversions pathway (‘-’ represents that it was not detected in mutant *msm2-1* or the wild type ‘FT’)

| Gene name | Gene locus | Up/Down | Log_2_FC | Annotation |
| --- | --- | --- | --- | --- |
| *PMEIs* | BraA03g023660.3C | Up | 8.83 | probable pectinesterase/pectinesterase inhibitor 17 |
|  | BraA05g004950.3C | Up | 9.91 | probable pectinesterase/pectinesterase inhibitor 17 |
|  | BraA03g032200.3C | Down | -13.83 | probable pectinesterase/pectinesterase inhibitor 21 isoform X1 |
|  | BraA02g040020.3C | Down | -13.26 | probable pectinesterase/pectinesterase inhibitor 58 |
|  | BraA09g005220.3C | Down | -12.78 | putative pectinesterase/pectinesterase inhibitor 28 |
|  | BraA06g033760.3C | Down | - | putative pectinesterase/pectinesterase inhibitor 28 |
|  | BraA01g041790.3C | Down | -13.06 | probable pectinesterase/pectinesterase inhibitor 23 |
|  | BraA08g011580.3C | Down | - | putative pectinesterase/pectinesterase inhibitor 43 |
|  | BraA01g042480.3C | Down | -5.23 | probable pectinesterase/pectinesterase inhibitor 21 |
|  | BraA05g038690.3C | Down | -9.93 | probable pectinesterase/pectinesterase inhibitor 23 |
| *PGs* | BraA04g031410.3C | Down | -7.99 | polygalacturonase-like |
|  | BraA08g024270.3C | Down | -13.13 | exopolygalacturonase clone GBGE184-like |
|  | BraA10g001430.3C | Down | -13.70 | exopolygalacturonase clone GBGE184-like |
|  | BraA08g024280.3C | Down | -13.63 | exopolygalacturonase clone GBGE184-like |
|  | BraA06g035800.3C | Down | -13.75 | polygalacturonase |
|  | BraA02g039380.3C | Down | -14.72 | polygalacturonase-like |
|  | BraA08g035590.3C | Down | -13.34 | exopolygalacturonase clone GBGE184-like |
|  | BraA02g039370.3C | Down | - | polygalacturonase-like |
|  | BraA02g039420.3C | Down | -12.91 | polygalacturonase-like |
|  | BraA02g039400.3C | Down | - | polygalacturonase-like |
|  | BraA02g039410.3C | Down | -12.62 | polygalacturonase-like |
|  | BraA09g065620.3C | Down | - | exopolygalacturonase clone GBGE184-like |
|  | BraA06g009640.3C | Up | - | putative pectate lyase 2 |
| *PLs* | BraA06g010580.3C | Down | -15.05 | probable pectate lyase 3 |
|  | BraA03g030560.3C | Down | -13.55 | probable pectate lyase 7 |
|  | BraA09g059390.3C | Down | - | probable pectate lyase 3 |
|  | BraA10g024240.3C | Down | -13.63 | probable pectate lyase 19 |
|  | BraA02g035760.3C | Down | -13.35 | probable pectate lyase 6 |
|  | BraA09g023600.3C | Down | -13.69 | pectate lyase-like protein 9 |
|  | BraA03g046540.3C | Down | -13.14 | probable pectate lyase 4 |
|  | BraA03g006620.3C | Down | -11.36 | probable pectate lyase 19 |
|  | BraA06g039690.3C | Down | - | probable pectate lyase 6 |
|  | BraA08g030210.3C | Down | -12.79 | probable pectate lyase 3 |

**Table S10** The different expression genes (DEGs) in the phenylpropanoid metabolism pathway (‘-’ represents that it was not detected in mutant *msm2-1* or the wild type ‘FT’)

| Key enzymes | Gene locus | Up/Down | Log_2_FC | Annotation |
| --- | --- | --- | --- | --- |
| Cinnamyl alcohol dehydrogenase | BraA01g004030.3C | Up | - | Cinnamyl alcohol dehydrogenase 5 |
|  | BraA09g055290.3C | Down | -6.34 | Cinnamyl alcohol dehydrogenase 2 |
| Feruloyl-CoA | BraA03g035560.3C | Down | -7.95 | Feruloyl CoA ortho-hydroxylase 2-like |
|  | BraA05g033360.3C | Down | -11.83 | Feruloyl CoA ortho-hydroxylase 2-like |
| Caffeoyl-CoA | BraA07g033240.3C | Down | -3.53 | Putative caffeoyl-CoA O-methyltransferase |
|  | BraA02g024180.3C | Down | - | Putative caffeoyl-CoA O-methyltransferase |

**Table S11** Differentially expressed genes in the pollen coat development of the wild-type ‘FT’ and *msm2-1* mutant (‘-’ represents that it is not detected in mutant *msm2-1* or the wild-type ‘FT’)

| Functional category | Gene name | Gene locus | Up/Down | Log_2_FC | Annotation |
| --- | --- | --- | --- | --- | --- |
| Pollen coat proteins  （*PCPs*） | GRPs | BraA10g029750.3C | Down | -12.38 | Encodes a glycine-rich protein that is expressed only in flowers during a specific developmental stage |
|  |  | BraA03g003050.3C | Down | -13.88 | GLYCINE RICH PROTEIN 17, encodes a glycine-rich protein that has an oleosin domain. |
|  |  | BraA03g003040.3C | Down | -12.69 | Encodes a glycine-rich protein that has an oleosin domain. |
|  |  | BraA03g003060.3C | Down | -5.72 | GLYCINE-RICH PROTEIN 19, a member of the oleosin-like protein family |
|  |  | BraA02g002380.3C | Down | -11.16 | GLYCINE RICH PROTEIN 17, encodes a glycine-rich protein that has an oleosin domain |
|  |  | BraA10g029710.3C | Down | -9.91 | GLYCINE-RICH PROTEIN 19, a member of the oleosin-like protein family |
|  |  | BraA03g003060.3C | Down | -5.72 | GLYCINE-RICH PROTEIN 19, a member of the oleosin-like protein family |
|  |  | BraA07g014240.3C | Down | - | Caleosin-related family protein |
|  |  | BraA02g002400.3C | Down | -7.11 | GLYCINE-RICH PROTEIN 20, lipid-binding oleosins, glycine-rich protein. |
|  | EXLs | BraA02g023130.3C | Down | -13.09 | EXL4; EXTRACELLULAR LIPASE 4, member of lipase proteins. |
|  |  | BraA02g023140.3C | Down | -13.62 | EXL6; EXTRACELLULAR LIPASE 6, member of lipase proteins. |
|  |  | BraA03g031210.3C | Down | -6.32 | EXL6; EXORDIUM LIKE 6 |
|  |  | BraA05g041570.3C | Down | -13.53 | EXL6; EXORDIUM LIKE 6 |
| Pollen coat lipids  （*PCLs*） | KCS7 | BraA02g020280.3C | No diff | * | A member of the 3-ketoacyl-CoA synthase family involved in the biosynthesis of VLCFA. |
|  |  | BraA07g029700.3C | No diff | * |  |
|  | KCS15 | BraA03g045900.3C | No diff | * | A member of the 3-ketoacyl-CoA synthase family involved in the biosynthesis of VLCFA. |
|  | KCS21 | BraA02g039980.3C | No diff | * | A member of the 3-ketoacyl-CoA synthase family involved in the biosynthesis of VLCFA. |

**Table S12** Differentially expressed genes in sporopollenin synthesis between the wild-type ‘FT’ and *msm2-1* mutant

| Functional category | Gene name | Gene locus | Up/Down | Log_2_FC | Annotation |
| --- | --- | --- | --- | --- | --- |
| Sporopollenin proteins  （*SSGs*） | ACOS5 | BraA09g012710.3C | Up | 16.73 | Encodes an acyl-CoA synthetase. Expressed in the tapetum and involved in pollen wall exine formation |
|  | MS2 | BraA01g039260.3C | Up | 12.72 |  |
|  | CYP703A2 | BraA09g066080.3C | Up | 8.68 | Member of CYP703A, CYP703A2 is expressed specifically in anthers, involved in pollen development |
|  | CYP704B1 | BraA07g034360.3C | Up | 13.26 | Encodes a cytochrome P450. Expressed in the developing anthers. Essential for pollen exine development |
|  | PKSA | BraA04g026900.3C | Up | 12.25 | POLYKETIDE SYNTHASE A, chalcone and stilbene synthase family protein |
|  | PKSB | BraA08g015650.3C | Up | 14.96 | POLYKETIDE SYNTHASE B, chalcone and stilbene synthase family protein |
|  |  | BraA01g003480.3C | Up | 11.77 |  |
|  |  | BraA03g059080.3C | Up | 12.34 |  |
|  | TKPR1 | BraA08g020830.3C | Up | 13.86 | TETRAKETIDE ALPHA-PYRONE REDUCTASE 1, encodes dihydroflavonol 4-reductase-like1, involved in a metabolic pathway essential for pollen wall development and male fertility |

**Table S13** Differentially expressed genes in tapetal development between the wild-type ‘FT’ and *msm2-1* mutant

| Functional category | Gene name | Gene locus | Up/Down | Log_2_FC | Annotation |
| --- | --- | --- | --- | --- | --- |
| Transcription factors | AMS | BraA07g004220.3C | Up | 2.66 | Encodes a basic helix-loop-helix transcription factor involved in tapetal cell development. |
|  |  | BraA03g043400.3C | Up | 2.16 |  |
|  | MS188 | BraA10g013970.3C | Up | 12.98 | Encodes a member of the R2R3 MYB transcription factor gene family that is required for the anther development by regulation tapetum development, callose dissolution and exine formation |
|  |  | BraA02g013010.3C | Up | 11.45 |  |
| Cysteine proteins | CEP1 | BraA03g026210.3C | Up | 4.09 | Encodes a papain-like cysteine protease involved in tapetal programmed cell death and pollen development. |
|  | CP1 | BraA10g004970.3C | Down | -8.57 | Cysteine peptidase, activity detected in leaf and flower. |
|  |  | BraA08g033980.3C | Down | -12.34 |  |
